# Delivery and Prioritization of Surgical Care in Canada During COVID-19: An Environmental Scan

**DOI:** 10.34172/ijhpm.2023.8007

**Published:** 2023-12-10

**Authors:** Seremi Ibadin, Mary Brindle, Tracy Wasylak, Jill Robert, Stacey Litvinchuk, Khara M. Sauro

**Affiliations:** ^1^Department of Community Health Sciences, Cumming School of Medicine, University of Calgary, Calgary, AB, Canada; ^2^Department of Surgery, Cumming School of Medicine, University of Calgary, Calgary, AB, Canada; ^3^Surgery Strategic Clinical Networks, Alberta Health Services, Calgary, AB, Canada; ^4^Surgery and Bone & Joint Health Strategic Clinical Networks, Alberta Health Services, Calgary, AB, Canada; ^5^Department of Oncology and Arnie Charbonneau Cancer Institute, Cumming School of Medicine, University of Calgary, Calgary, AB, Canada

**Keywords:** Surgery, COVID-19 Pandemic, Policy, Surgical Backlog, Surgical Waitlist, Canada

## Abstract

**Background:** During COVID-19 healthcare systems had to make concessions to make room for the surge of COVID-19 patients requiring hospital and intensive care. Postponing surgeries was a common strategy; however, it is unclear how surgical care was delivered during this time of constraint. The objective of this study was to understand how surgical care was delivered and prioritized during the COVID-19 pandemic response.

**Methods:** This was an environmental scan following the Canadian Agency for Drugs and Technologies in Health methodology. This study was conducted in Canada; a universal, publicly funded healthcare system. Evidence sources on policies pertaining to the provision of surgical care between January 2020 and October 2022 were obtained from ministries of health, health services agencies and publicly funded hospitals across all 10 provinces and three territories. We synthesized the evidence sources using framework analysis.

**Results:** We identified 205 evidence sources that described six themes about the provision of surgical care during the COVID-19 pandemic: the cycle of postponement and resumption; guidelines for triaging and prioritizing surgical cases; Infection Prevention and Control (IPAC), and safety measures for surgical care during COVID-19, patient-centred care, and looking forward (recovery planning, leadership, and decision-making).

**Conclusion:** This study provides a comprehensive understanding of how surgical care was disrupted and innovated during COVID-19 which can inform future strategies for providing effective and efficient surgical care during times of healthcare constraint.

## Background

**Key Messages**

**Implications for policy makers**

- This study provides policy-makers with an understanding how surgical care was provided during COVID-19 and efforts to increase surgical capacity in the wake of COVID-19, which can inform future policies for surgical care during times of healthcare constraint and how to rebuild surgical capacity.
- Because of the urgent and evolving nature of COVID-19 many policy-makers were required to rapidly make decisions with little evidence or time to consult with policy-makers in other jurisdictions, many policies and their rollout differed between jurisdictions and were done in isolation.
- This study was co-developed by policy-makers to elucidate surgical policies across a large, universal, publicly funded healthcare system during a public health crisis.

**Implications for the public**

This study sought to understand how surgical care was delivered during the COVID-19 pandemic response. Based on the 205 evidence sources included in our analysis, we described the cycle of postponement and resumption; guidelines for triaging and prioritizing surgical cases; Infection Prevention and Control (IPAC), and safety measures for surgical care during COVID-19, patient-centred care, and looking forward (recovery planning, leadership, and decision-making). This study provides the public with an understanding of how surgical care was disrupted during COVID-19 and how these decisions were made. This study also highlights how the healthcare system is trying to overcome the backlogs created by disruptions to surgical care during COVID-19.

The COVID-19 pandemic and the response to it, strained health systems globally resulting in unprecedented changes to the delivery of health services.^1-3^ Like other countries, Canadian healthcare systems were suddenly tasked with curbing the spread of COVID-19 and caring for the surge of patients with COVID-19, while simultaneously ensuring the healthcare needs of the general population were being met. One strategy that was used, was postponing non-urgent scheduled surgeries to make room for the increasing number of patients with COVID-19 requiring hospitalization and intensive care. Postponing non-urgent surgeries during the pandemic response resulted in surgical backlogs and increased wait times for surgery; almost 600 000 fewer surgeries were performed across Canada between March 2020 and December 2021, compared to 2019 surgical volumes.^4^

 It is estimated that the backlog of surgeries created from the COVID-19 pandemic response will take years to clear.^5,6^ The impact of delaying non-urgent surgeries is widespread. Studies exploring the impact on patients who had their surgeries cancelled or delayed showed that patients experienced a great deal of distress, with many suggesting both their physical and mental health suffered.^7,8^ The distress of delaying non-urgent surgeries extended to surgeons who worried about their patients’ health, had to shift responsibilities away from surgery to help with the COVID-19 response, the consequences of which extended into their personal lives.^9^ There were also wider impacts of the COVID-19 response; it has been reported that there has been an excessive death rate (beyond COVID-19 cases) in several health conditions some of which may be directly related to delayed surgeries,^10-12^ and fewer patients sought medical care for fear of contracting COVID-19 in healthcare settings which is likely to have a trickle-down effect for some time.^13,14^ Despite all that is now known about the impact of the COVID-19 response, during the height of COVID-19 little evidence was available to support decision-making.

 At the beginning of the pandemic, across the world, there was a rapid mobilization of experts to create guidelines and recommendations to guide surgical care. These recommendations had three main foci: prioritize surgeries based on the most acutely needed surgeries, outline strategies to safely perform surgeries to prevent the spread of COVID-19, and how to increase the number of non-urgent surgeries being completed without overwhelming hospitals during COVID-19.^6,15-21^ Several similar guidelines were developed within the Canadian context.^15,17,19^ As the pandemic evolved, research and recommendations focused more on guiding surgical recovery efforts.^5,21-23^ However, anecdotal evidence suggests that there was no consensus or universally adopted approach for managing surgical care across Canada, and there has been little cross-jurisdictional interaction towards planning the way forward for surgical care in Canada. This may be a missed opportunity to learn from successes and challenges during the COVID-19 pandemic and in other times of constrained healthcare resources.

 Although there has been research on surgical care during COVID-19, to our knowledge there does not appear to be a research study that comprehensively describes how surgical care was operationally prioritized and delivered across a country. The objective of this study was to describe surgical care during COVID-19 across Canada, mapped to COVID-19 metrics. Understanding how surgical care was delivered across Canada during COVID-19 is crucial to support recovery efforts and preparedness for future public health emergencies.

## Methods

###  Study Setting

 Healthcare in Canada, including surgical care, is universal and publicly funded. Funding for healthcare is transferred from the federal government to provincial and territorial governments to deliver healthcare services. All provinces and territories manage the delivery of surgical care through their Ministries or Departments of Health and provincial or regional health authorities.^24^ Surgeries in Canada are predominantly performed at public healthcare facilities. However, some provinces have contractual agreements with private surgical facilities to deliver selected publicly funded surgical procedures at private facilities.^25^

###  Study Design

 We used environmental scan methodology to describe surgical care policies across Canada during the COVID-19 response (March 2020 until October 2022), using the methods outlined by the Canadian Agency for Drugs and Technologies in Health.^26,27^ Using this methodology, evidence sources that describe surgical care policies across Canada were collected and analyzed using the methods described below.

###  Search Strategy

 Using a three-step process we searched for evidence sources. In the first step, we searched the internet for documents that contained surgical care policies created in response to COVID-19 using search terms for COVID-19 (eg, COVID, coronavirus, and SARS-CoV-2), policy (eg, guidelines, guidance, recommendation, and protocol) and surgical care (eg, surgery, operations, procedures, non-urgent, elective, and scheduled). This initial search was conducted on Google search engine and websites of ministries of health, provincial and regional health authorities and publicly owned healthcare facilities with surgery departments across Canada (guided by a list retrieved from the Canadian Institute for Health Information website).^28^ Next, we screened the initial evidence sources to refine our search terms; we identified terms related to delays, priority, screening, testing, infection prevention, safety, backlogs, recovery which were used to conduct a more focused search of websites and reference lists of related publications to identify additional evidence sources (snowball sampling). Finally, we contacted operational and clinical leads in surgical care (eg, department heads, chiefs of surgery, and operational directors) using emails and through our research team to retrieve documents that were not publicly available.

###  Eligibility Criteria 

 Evidence sources were included if they met all the following criteria: (1) were policy documents (including policies, guidance, protocols, plans, memos, technical reports, official news updates, and information materials) that communicated policies for the provision of surgical care, including reorganization of surgical services during COVID-19; (2) were written by health regulatory authorities (ministries and local health authorities), leadership of healthcare facilities, and professional bodies (College of Surgeons and Provincial Medical Associations) and (3) were made available to those delivering surgical care (published publicly or circulated within relevant institutions or departments) between January 2020 and October 2022. To ensure credibility of evidence sources, news articles, blog posts, opinion articles and commentaries that were not official positions of provincial, regional, and local health authorities or healthcare facilities were excluded. Diagnostic and therapeutic guidelines published within the study period that did not specifically describe changes in surgical care due to COVID-19 were excluded, as they are beyond the scope of this study. We also excluded duplicate documents or correspondences; evidence sources were considered duplicate if they were redistributions of previous evidence sources within the same province and did not include additional information when compared with the original evidence source (eg, updates to confirm policies remained in place).

###  Data Extraction 

 We used a standardized data abstraction form in Microsoft Excel to extract data from all included evidence sources along with a data dictionary which defined the data elements (Supplementary file 1). This form captured document characteristics (title, author information, date, and type of documents) and policy-specific data (policy problem, objective, category, content description, etc). After pilot testing the form using a sample of 40 evidence sources, we revised the abstraction form accordingly to ensure comprehensiveness, relevance, and reliability. Data was abstracted by a single reviewer with consultation by a second reviewer.

###  Qualitative Interviews

 For provinces and territories where evidence sources were scarce, we conducted interviews with healthcare leaders to understand the approaches taken for delivering surgical care during COVID-19, using a semi-structured interview guide. Interview participants were purposively selected based on their involvement in policy development, their direct experience with the operationalization of these policies, and their capacity to provide informed opinions relevant to the subject matter. Interviews were conducted virtually by a trained research associate and recorded with the consent of participants. The interviews were audio recordings and transcribed verbatim.

###  Epidemiologic Data

 We retrieved national data on the number of COVID-19 cases, hospitalizations, intensive care unit (ICU) admissions and deaths from January 18, 2020 to October 29, 2022, from the Government of Canada’s COVID-19 epidemiology web page^29^ to contextualize the data obtained from the evidence sources.

###  Data Analysis

####  Data Analysis of the Evidence Sources and Interviews

 Characteristics of the included evidence sources were summarized as counts. Qualitative data from the included evidence sources were imported into NVivo-12 (QSR International, Melbourne, Australia) for data management and analysis. We applied framework analysis (combining deductive and inductive approaches) to synthesize the data into overarching themes and subthemes, providing a structured summary of the data.^30^ This process entailed six stages: (1) familiarization with evidence sources (one analyst read through all the evidence sources to gain an overview of the data and become well-acquainted with its content); (2) open coding (the analyst generated initial codes using predetermined concepts deduced from existing literature and new concepts that emerged from preliminary coding on a sample of 20 evidence sources); (3) developing an analytical framework (codes were revised with the team, sorted and grouped together based on emerging patterns to create themes and subthemes, and the framework for analysis was generated); (4) applying the analytical framework to remaining evidence sources (we coded the remaining evidence sources using the developed framework and continually added new codes as they emerged); (5) charting data into the framework matrix (ie, mapping data from each evidence source to the framework); and (6) interpreting the data. Data analysis was an iterative process that continued until completion. Codes and themes were documented in a codebook to enable research team members to crosscheck and confirm findings.

 Prior to analysis, we reflected on how our professional roles, experiences and ideas may influence our findings. The primary analyst was a public health physician who had not practiced in Canada during the study and the secondary analyst was the Principal Investigator of the study. To maintain neutrality, only members of the research team who did not participate in developing, implementing, or evaluating surgical care policies (in provincial, regional, or local health authority or surgery department across Canada) were involved with data extraction and analysis processes.

####  Data Analysis of Epidemiological Data 

 COVID-19 cases, hospitalizations, ICU admissions and deaths were synthesised as counts and were plotted as overlapping line graphs.

 Findings from the qualitative analysis of the evidence sources were interpreted within the context of COVID-19 epidemiologic data. This triangulation enabled a well-rounded understanding of surgical care policies throughout COVID-19.

## Results

###  Search Results and Document Characteristics

**Figure 1:**
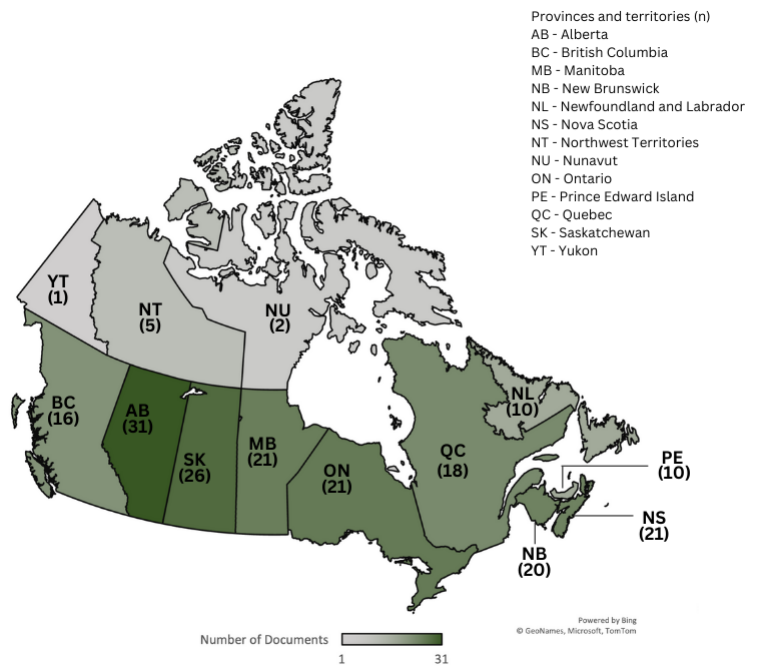
Number of Evidence Sources Included by Province and Territory.

 We analyzed 205 evidence sources from all 10 provinces and three territories and included data from four interviews with surgical care leaders across Canada (Figure 1).

Table 1 summarizes the types of evidence sources included and detailed characteristics of all evidence sources are contained in Table S1 (Supplementary file 2).

**Table 1:** Type of Evidence Sources Included Across Canada

**Jurisdiction**	**Document Type **						**Total **
	**Guideline **	**Memo **	**Plan **	**Report (News Release)**	**Report (Technical) **	**Informational/FAQs **	
**Province**							
AB	6	8	7	7	3	0	31
BC	4	0	2	8	0	2	16
MB	2	0	2	16	1	0	21
NB	0	0	1	15	2	2	20
NL	0	0	1	8	1	0	10
NS	2	0	4	11	3	1	21
ON	5	9	2	1	0	4	21
PE	5	1	1	0	3	0	10
QC	7	11	0	0	0	0	18
SK	11	5	3	3	2	2	26
**Territory**							
NT	0	0	0	4	0	1	5
NU	0	0	0	2	0	0	2
YT	0	0	0	0	0	1	1
**Federal **	0	0	0	2	1	0	3
**Total**	42	34	23	77	16	13	205

Abbreviations: AB, Alberta; BC, British Columbia; MB, Manitoba; NB, New Brunswick; NL, Newfoundland and Labrador; NS, Nova Scotia; ON, Ontario; PE, Prince Edward Island; QC, Quebec; SK, Saskatchewan; NU, Nunavut; YT, Yukon; NT, Northwest Territories; FAQs, frequently-asked questions.

###  Qualitative Data on Policies for Provision of Care

 We identified six themes related to the evolution of surgical care policies during COVID-19: the cycle of postponement and resumption; guidelines for triaging and prioritizing surgical cases; Infection Prevention and Control (IPAC), and safety measures for surgical care during COVID-19, patient-centred care, and looking forward – surgical recovery planning, leadership and decision-making (Table 2).

**Table 2:** Summary of Reorganization Strategies Across Canada

**Themes**	**Province**										**Territory**		
	**AB**	**BC**	**MB**	**NB**	**NL**	**NS**	**ON**	**PE**	**QC**	**SK**	**NT**	**NU**	**YT**
**Theme 1: Cycle of postponement and resumption of non-urgent surgeries**^a^	✓	✓	✓	✓	✓	✓	✓	✓	✓	✓	✓	✓	✓
**Theme 2: Guidelines for triaging and prioritization of surgical cases**													
Had documented prioritization guidelines	✓	✓	✓			✓	✓	✓	✓	✓			
Had specific cancer surgery prioritization recommendations	✓	✓				✓	✓		✓	✓			
Guideline recommended pre-determined level of surgical activity and reduction in non-urgent surgery based on pandemic response phase or level	✓		✓				✓		✓				
Non-urgent surgeries prioritized using categorization or coding system	✓		✓				✓		✓				
Guideline recommended use of triage bands based on American College of Surgeons recommendation						✓		✓					
Used algorithm to guide surgical prioritization based on trigger factors										✓			
**Theme 3: IPAC and safety measures for surgical care**													
Had documented IPAC protocols or safety measures for surgery during COVID-19	✓	✓	✓	✓		✓	✓	✓	✓	✓			
Had information on COVID-19 screening, testing and recommendations on preparing for surgeries among patients with COVID-19	✓	✓	✓	✓		✓	✓	✓	✓	✓		✓	
Recommended patients booked for surgery should limit exposure risk	✓	✓	✓	✓		✓	✓	✓	✓	✓			
Patients booked for surgery recommended to self-isolate		✓		✓		✓	✓						
Patients instructed to conduct pre-operative screening and risk assessment	✓	✓	✓	✓		✓	✓	✓	✓	✓			
Patients recommended to use virtual visits for pre-admission clinics to minimize contact where available	✓	✓	✓				✓						
Pre-operative testing required							✓		✓	✓			
Pre-operative testing may be required based on screening and risk assessment	✓	✓				✓							
Pre-operative testing during high transmission, risk-based testing during reduced transmission			✓									✓	
Voluntary testing for all surgical patients											✓		
Documents described recommendations on timing of non-urgent surgery after COVID-19 infection	✓	✓		✓		✓			✓	✓	✓		
Recommendation to defer non-urgent surgery for up to 7 weeks after COVID-19 infection	✓									✓^b^			
Recommendation to defer non-urgent surgery for up to 3 to 4 weeks after COVID-19 infection									✓	✓			
Recommendation to defer non-urgent surgery for up to 10 to 14 days after COVID-19 infection				✓		✓							
Recommendation to defer non-urgent surgery based on clinical judgment, no specific guidance on timing		✓									✓		
Documents described changes in IPAC protocols for peri-operative teams	✓	✓				✓	✓	✓	✓	✓			
Documents described changes in OR utilization	✓	✓		✓		✓	✓	✓	✓	✓			
**Theme 4: Patient-centred care**													
Documents described communication with patients	✓	✓	✓	✓	✓	✓	✓	✓	✓	✓	✓	✓	✓
Documents described patient involvement in decision-making beyond individual care decisions	✓		✓										
Provided information instructing patients with delayed surgeries should contact their doctor's office, call helplines or visit the Emergency Room as appropriate	✓	✓	✓	✓	✓	✓	✓	✓	✓	✓	✓	✓	✓
**Theme 5: Looking Forward - Surgical Recovery Plans**													
Use designated surgical sites within regions	✓		✓							✓			
Use virtual clinics for peri-operative care	✓	✓	✓				✓						
Create/implement centralized waitlist management system	✓	✓				✓	✓						
Create/implement centralized single-entry referral	✓	✓				✓							
Increased OR hours	✓	✓	✓						✓	✓			
Increased publicly funded surgeries at private facilities within province	✓	✓	✓				✓		✓	✓			
Create new, and expand existing ORs	✓	✓	✓			✓				✓			
Maximize available surgical bed capacity by improving care pathways	✓	✓				✓	✓						
Convert eligible procedures to day surgeries	✓	✓			✓	✓							
Contract out-of-province facilities for eligible cases			✓		✓					✓			
Increase recruitment and training of health workers	✓	✓	✓						✓	✓			
Use existing health workers in new roles to support surgical services	✓												
Ensure critical supplies	✓	✓	✓	✓	✓	✓	✓	✓	✓	✓	✓	✓	✓
Funding – Federal allocation to clear surgical backlogs	✓	✓	✓	✓	✓	✓	✓	✓	✓	✓	✓	✓	✓
Funding – provincial budget allocation and investment	✓	✓	✓		✓	✓	✓		✓	✓			
Funding – private investments and donations			✓										
**Theme 6: Leadership and decision-making**	✓	✓	✓	✓	✓	✓	✓	✓	✓	✓	✓	✓	✓

Abbreviations: AB, Alberta; BC, British Columbia; MB, Manitoba; NB, New Brunswick; NL, Newfoundland and Labrador; NS, Nova Scotia; ON, Ontario; PE, Prince Edward Island; QC, Quebec; SK, Saskatchewan; NU, Nunavut; YT, Yukon; NT, Northwest Territories; IPAC, Infection Prevention and Control; OR, operating room. ^a^ Cycle of postponement and resumption is depicted in Figure 2. ^b^ Recommended for asymptomatic patients in SK.

###  Theme 1: The Cycle of Postponement and Resumption 

**Figure 2:**
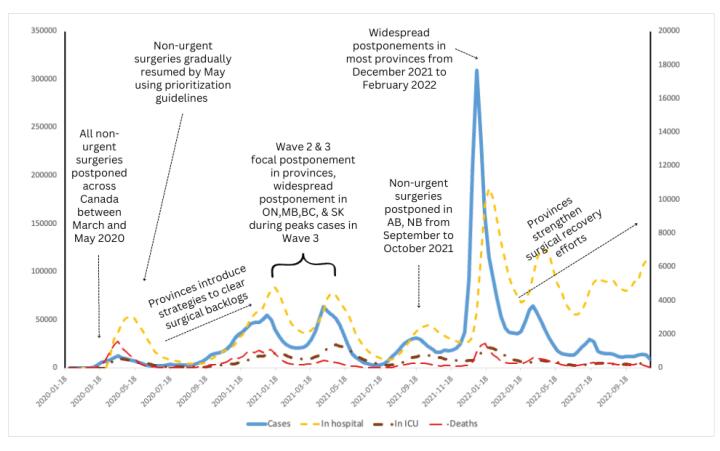
Timeline for Postponement and Resumption of Non-urgent Surgeries Across Canada During COVID-19. Abbreviations: AB, Alberta; BC, British Columbia; MB, Manitoba; NB, New Brunswick; ICU, intensive care unit; SK, Saskatchewan; ON, Ontario.

 Seventy evidence sources from all provinces and territories contained information about postponing or resuming non-urgent surgeries. Over the first 24 months of the pandemic, all provinces and territories at some point implemented policies to postpone non-urgent surgeries. All provinces and territories also had policies for resuming non-urgent surgeries, and 10 provinces and territories (Alberta [AB], British Columbia [BC], Manitoba [MB], New Brunswick [NB], Nova Scotia [NS], Nunavut [NU] Ontario [ON], Prince Edward Island [PE], Quebec [QC], and Saskatchewan [SK]) had guidance for returning to normal levels of surgical capacity. The timeline for implementation of these policies across Canada is depicted in Figure 2.

 Two sub-themes emerged around the cycle of postponement and resumption of surgeries.

####  1. Approaches for Postponing and Resuming Scheduled Non-urgent Surgeries

 Provinces adopted two main approaches to postponing scheduled non-urgent surgeries: either province-wide postponements (cessation of all scheduled non-urgent surgeries) or focused surgical postponements (ie, regional or site-specific reduction of scheduled non-urgent surgeries). In the first wave of COVID-19, province-wide postponements occurred in all provinces, and by the second wave, focal surgical postponements were more predominantly used. In the third wave, province-wide postponements re-occurred in four provinces (BC, MB, ON, and SK). By the fourth and fifth waves, most provinces had some postponements. During the sixth wave, no provinces strategically reduced surgical volume (Figure 2). Following postponements, nine provinces had guidelines for gradual or staged resumption of surgeries (AB, BC, MB, NB, NS, ON, PE, QC, and SK) based on some form of prioritization (described in Theme 2 – Guidelines for triaging and prioritizing surgical cases).

####  2. Rationale for Postponing and Resuming Non-urgent Surgeries 

 The main reasons for postponing scheduled non-urgent surgeries during COVID-19 were to: implement directives from provincial Chief Medical Officer of Health, preserve acute care resources (eg, hospital and ICU beds, staff and supplies) to care for the surge in patients with COVID-19, ensure the safety of patients and health workers (ie, prevent COVID-19 infection), and respond to workforce shortage owing to illness and absences among health workers.

 On the other hand, the rationale for deciding to resume scheduled non-urgent surgeries included: growing surgical backlogs, concerns for patient health and well-being, declining COVID-19 transmission, and ability to dedicate healthcare resources for surgical care. Furthermore, evidence sources from five provinces (AB, BC, ON, QC, and SK) indicated that resumption of non-urgent surgeries was conditional upon certain requirements; ethical principles (equity, proportionality, non-maleficence, and reciprocity) and operational requirements (availability of guidelines for triaging and prioritization, sufficient capacity to accommodate COVID-19 surges, adequate availability of critical supplies, capacity for pre- and post-operative care, and effective monitoring to inform further decision-making).

###  Theme 2: Guidelines for Triaging and Prioritizing Surgical Cases

 Surgery postponements in the first wave of COVID-19 prompted the development and use of guidelines to facilitate prioritizing surgeries during periods of constraint. Thirty-one evidence sources from eight provinces (AB, BC, MB, NS, ON, PE, QC, and SK) contained guidance on surgical prioritization (Table 3 and Table S2 of Supplementary file 3). Although prioritization approaches varied, urgency of the surgery was universally the single most important criteria. Prioritization guidance also considered disease factors (severity, risk of progression/complications while waiting for surgery), patient factors (age, comorbidities), duration on surgical waitlist and resource requirements for surgery and recovery (eg, day surgeries or clinic-based procedures vs surgeries requiring inpatient admission or ICU care).

**Table 3:** Approaches to Prioritizing Surgery During COVID-19

**Guidance for Prioritizing Surgery During COVID-19 (Overall)**	**Provinces**
Pre-determined level of surgical activity and reduction in non-urgent surgeries based on pandemic response level or phase	AB, MB, ON, QC
Non-urgent surgeries prioritized using categorization or coding system based on wait times	
No pre-determined level of surgical activity based on pandemic response phase	NS, PE
Recommended use of Triage Bands based on American College of Surgeons recommendations	
Algorithm to guide surgical prioritization decisions based on trigger factors	SK

Abbreviations: AB, Alberta; MB, Manitoba; NS, Nova Scotia; ON, Ontario; PE, Prince Edward Island; QC, Quebec; SK, Saskatchewan.

 Cancer surgeries were prioritized throughout the pandemic in all provinces and cancer surgery prioritization recommendations were specifically developed for the COVID-19 pandemic in six provinces (AB, BC, NS, ON, QC, and SK). These recommendations considered type and stage of cancer, risk of progression, treatment intent and availability of therapeutic alternatives to determine which cancer surgeries could proceed (or could be delayed) during periods of surgical postponement (Table S2).

###  Theme 3: Infection Prevention and Control, and Safety Measures for Surgical Care During COVID-19

 Forty-six evidence sources from nine provinces (AB, BC, MB, NB, NS, ON, PE, QC, and SK) documented IPAC protocols or safety measures for surgical care during COVID-19 for patients and healthcare workers. Key findings are summarized into three sub-themes.

####  a. COVID-19 Risk – Protocols and Testing 

 Twenty-two evidence sources from ten provinces and territories (AB, BC, MB, NB, NS, NU, ON, PE, QC, and SK) had information on COVID-19 screening, testing and recommendations on preparation for surgeries among patients with COVID-19. Patients booked for surgery during COVID-19 pandemic were advised to limit COVID-19 exposure risk in nine provinces for which there were evidence sources (AB, BC, MB, NB, NS, ON, PE, QC, and SK) and self-isolate prior to surgery (recommendations varied between 10 to 14 days) in four provinces (BC, NB, NS, and ON). Patients were also instructed to conduct pre-operative screening and risk assessment (AB, BC, MB, NB, NS, ON, PE, QC, and SK) and use virtual visits for pre-admission clinics to minimize contact where available (AB, BC, ON, and MB). The main approaches to pre-operative COVID-19 testing are listed in Table 4.

**Table 4:** Pre-operative COVID-19 Testing Requirements and Timing of Elective Surgeries After COVID-19

**Pre-operative COVID-19 Testing Approach**	**Provinces and Territories**
Pre-operative testing required	ON, QC, SK
Pre-operative testing may be required based on screening and risk assessment	AB, BC, NS
Pre-operative testing required during periods of high transmission Risk-based testing used during periods of reduced transmission	MB, NU
Voluntary testing for all surgical patients	NT
**Recommended Timing for Elective Surgery After COVID-19**	
Defer non-urgent surgery for up to 7 weeks	AB, SK (symptomatic patients)
Defer non-urgent surgery for up to 3 to 4 weeks	QC, SK (asymptomatic patients)
Defer non-urgent surgery for 10 to 14 days	NB, NS
Defer non-urgent surgery based on clinical judgement, no specific guidance provided	BC, NT

Abbreviations: AB, Alberta; BC, British Columbia; MB, Manitoba; NB, New Brunswick; NS, Nova Scotia; ON, Ontario; QC, Quebec; SK, Saskatchewan; NU, Nunavut; NT, Northwest Territories.

 Among patients with COVID-19, emergent or urgent surgeries could proceed in accordance with the recommended IPAC protocol. Non-urgent surgeries were scheduled based on clinical judgment and provincial guidelines for timing of elective surgeries after COVID-19 (Table 3).

####  b. Changes in IPAC Protocols for Perioperative Teams 

 Among seven provinces (AB, BC, NS, ON, PE, QC, and SK), 24 evidence sources described clinical pathways for surgical patients (based on COVID-19 exposure risk or status) that enabled surgical teams to adopt required IPAC protocols to minimize risk to healthcare providers and other patients. Changes to IPAC protocols that were implemented during COVID-19 included; modifying the use of personal protective equipment based on point-of-care risk assessment, limits to the number of staff required for surgery or procedures (eg, surgical team may be required to stay outside the OR when the anesthetic team is intubating and extubating), minimizing required equipment, minimizing traffic, regulating door opening and controlling airflow (exchanges, temperature, humidity) in the OR. A list of IPAC guidance is included in Table S3 (Supplementary file 4).

####  c. Changes in OR Utilization 

 Twenty-four evidence sources provided guidance on changes to OR utilization to reduce COVID-19 infection. These recommendations varied by province and included the use of dedicated ORs for COVID-19 positive cases (AB, NB, NS, QC, and PE), scheduling COVID-19 cases as the last procedure for the day or at night (AB), use of positive pressure ORs (AB) or positive pressure ORs with negative-pressure anterooms (BC, QC, and SK) or negative pressure ORs (PE and QC), and changes to scheduling taking into consideration the additional time for enhanced cleaning and disinfection procedures. Provinces revised their policies as evidence emerged about the mechanisms of COVID-19 transmission. For example, Saskatchewan dismantled the temporary negative pressure anterooms erected earlier (June 2020) when new evidence emerged. Alberta’s updated guidelines (May 2021) allowed for the use of any OR for COVID-19 cases, rather than designating COVID-positive ORs.

###  Theme 4: Patient- Centred Care

 Eighty-four evidence sources obtained from all provinces and territories included policies that acknowledged the concept of patient-centred care,^31,32^ for which four sub-themes emerged.

####  1. Communication With Patients 

 In all provinces and territories, direct communication with patients about their surgical care was noted to be primarily through their surgeon’s office. Communication included discussions about postponements, rescheduling, and preparation for surgery. Provincial health authorities and facilities also published information for surgical patients on their websites in all provinces.

####  2. Patient Involvement in Decision-Making

 Two provinces (AB and MB) documented the inclusion of patients’ voices in their surgical recovery planning process (the leadership of the Alberta Surgical Initiative consulted with patient groups and Manitoba Government appointed patient and citizen representatives as members of the Diagnostic and Surgical Recovery Task Force). Apart from discussions about individual care, there were no evidence sources that included patients or caregivers in facility, regional or provincial/territorial decision-making regarding postponement of scheduled non-urgent surgeries and prioritization of surgical cases during COVID-19.

####  3. Managing Patients With Delayed Surgeries 

 In all provinces and territories, patients waiting to have their surgeries rescheduled were advised to contact their family physician or surgeon’s office, call help lines, or go to the emergency department if they had worsening symptoms or health problems. We did not find additional information or resources tailored to patients experiencing surgery delays.

####  4. Concerns About the Impact of Delayed Surgeries on Patients 

 All provincial and territorial governments acknowledged that surgery delays may be associated with negative impacts on patients’ health and quality of life and concerns for patients were considered when developing plans to reduce surgical backlogs and achieve recommended wait times.

###  Theme 5: Looking Forward - Surgical Recovery Planning

 Fifty-two evidence sources from all provinces and territories discussed surgical recovery planning. Surgical recovery planning commenced in the first wave, as non-urgent surgeries resumed, albeit to varying degrees across provinces. In general, the documented recovery plans aimed to clear surgical backlogs and ensure Canadians receive surgery within acceptable wait times based on Canadian Institute for Health Information benchmarks for recommended wait times. The strategies implemented immediately after the strategic postponement of non-urgent surgeries to clear backlogs were increasing operating room (OR) hours on weekdays, scheduling surgeries on weekends and designating facilities to be used as dedicated surgical sites. Medium to long-term strategies focused on increasing surgical capacity by contracting private surgical clinics and out-of-province facilities to complete more surgeries, building new ORs, expanding existing ORs and ICU beds at public facilities, increasing human resources (hiring more doctors, nurses, and allied healthcare providers) and improving efficiency (develop centralized waitlist management systems, single-entry referral systems, improve care pathways and transition to long term care settings). Funding commitments were made by the Federal Government ($4 billion in 2021 and $2 billion in 2022) and provincial governments to clear surgical backlogs. Table 5 summarizes the main strategies for surgical recovery across provinces, and Table S4 of Supplementary file 5 provides details on strategies, targets, funding commitments and progress.

**Table 5:** Summary of Provincial Strategies for Clearing Surgical Backlogs/Surgical Recovery

**Category **	**Strategies **	**Provinces **
Increase efficiency and leverage technology	Use designated surgical sites within regions	AB, SK, MB
	Use virtual care clinics for peri-operative care	AB, BC, ON, MB
	Data analytics/modelling to aid decision-making on surgical capacity vs COVID-19 surge capacity	AB, ON, BC, NS
	Create/implement centralized waitlist management system	AB, BC, ON, SK, NS
	Create/implement centralized single-entry referral system	AB, BC, NS
Increase surgical capacity	Increase OR hours (longer hours during weekdays, surgeries scheduled on weekends, rearranging vacation time, cutbacks on research and academic work)	AB, BC, SK, MB, QC
	Increase publicly funded surgeries at private facilities within the province	AB^a^, BC, SK, MB, ON, QC^b^
	Create new, and expand existing ORs	AB, BC, SK, MB, NS
	Maximize available surgical bed capacity (improve surgical care pathway to reduce length of in-patient stay, improve transition to long-term facilities and community care)	AB, NS, ON, BC
	Convert eligible procedures to day surgeries where feasible	AB, BC, NS, NL
	Contract out-of-province facilities for eligible cases	SK,^c^ MB,^d^ NL^e^
	Increase recruitment and training of health workers	AB, SK, BC, MB, QC
	Use existing health workers in new roles to support surgical services	AB^f^
Ensure critical supplies	Adequate stock of PPE, disinfection supplies	All provinces
Ensure funding	Federal allocation to clear surgical backlogs	All provinces
	Provincial budget allocation and investments	AB, BC, MB, NS, SK, NL, ON, QC
	Private investments and donations	MB

Abbreviations: AB, Alberta; BC, British Columbia; MB, Manitoba; NL, Newfoundland and Labrador; NS, Nova Scotia; ON, Ontario; QC, Quebec; SK, Saskatchewan; PPE, personal protective equipment; OR, operating room. ^a^Orthopaedic and ophthalmology; ^b^Orthopaedic; ^c^20 to 25 knee and hip replacements surgeries per month performed in private facility in Alberta by fall 2022; ^d^Orthopedic (hip, knee and spinal) surgeries; performed in Ontario (CA), Ohio and North Dakota (USA) by 2022; ^e^Partnership with University of Ottawa Heart Institute to perform cardiac surgeries; ^f^Alberta Health Services piloted Anesthesia Care Team model that allows respiratory therapists support anesthesiologist during cataract surgeries that require topical or minimal sedation in 2022.

###  Theme 6: Leadership and Decision-Making

 Overall, we observed that policies were formulated through dynamic and consultative processes led by provincial governments. Surgical policies were mainly formulated at the provincial level, spearheaded by clinical and health system operational leaders. Decision-making involved consideration of policy options using multiple evidence sources (ie, expert opinion, jurisdictional scans, and reviews of international evidence and provincial data). Policies were adopted based on expert recommendations or consensus (all provinces had established at least one expert committee, working group or task force to support decision-making). Provincial guidelines were disseminated across health regions and facilities for implementation. At the regional or facility level, committees used local epidemiologic conditions and assessment of surgical capacity to operationalize policies in their surgery departments. Within surgical departments, surgeons relied on clinical judgement and guidelines to reach decisions regarding individual patient care. Patient involvement in decision-making has been previously highlighted. Continuous feedback was used to evaluate policies and generate evidence to support maintenance or revision of policies.

## Discussion

 This study found that policies about surgical care during COVID-19 across Canada mainly focused on decisions to postpone, prioritize, or resume non-urgent surgeries, and limiting COVID-19 risk for patients and healthcare providers. Policies guiding surgical care evolved as did the process for making policies during COVID-19. Policies were broadly similar across provinces in their overarching objectives; however, there were variations in specific surgical prioritization guidelines, IPAC recommendations and provincial plans to manage surgical backlogs and improve wait times for surgery. The surgery delays during COVID-19 created an impetus for governments to quickly fund interventions to increase efficiency and expand capacity for surgical care, which will impact surgical care beyond COVID-19.

 Postponing non-urgent surgery was a strategic measure to ensure that healthcare systems were not overwhelmed during the pandemic.^33,34^ Consequently, surgical patients became collateral damage in mitigating the impact of the COVID-19 pandemic. Globally it is estimated that 28 million surgeries were cancelled in the first three months of the pandemic alone.^35^ In Canada, studies and local health authorities suggest that surgical volume was reduced to 60%-70% of normal volume.^4,13,28^ The disruption to surgeries being completed was differential between types of surgeries – ophthalmological and orthopedic surgeries were most likely to be cancelled or postponed.^13,36,37^ Other studies suggest that while cancer surgeries continued, diagnostic surgical services required for cancer detection and staging were negatively impacted by delays and may create a greater need for cancer surgeries beyond COVID-19.^38,39^ These findings are aligned with the policies identified in the present study, where certain surgeries were prioritized such as cancer surgeries. Collectively, this highlights the important impact that health policy has on healthcare delivery.

 Given the impact that policies have on healthcare delivery, it is important to ensure that implemented policies are evidence-based and address the needs of the population. This was true during COVID-19 but also beyond COVID-19. However, the objective and desired outcomes of policies are different beyond COVID-19; to increase surgical capacity rather than decrease it. Some evidence-based strategies for reducing surgery wait times include outsourcing publicly funded surgeries to private surgical facilities,^23,37,40^ central referral and waitlist management systems,^23,41,42^ implementing surgical pathways,^43-45^ and creating a larger role for family physicians and allied healthcare providers.^23,40,46,47^ While these strategies have some evidence to support their effectiveness,^21,23,40,41,43-50^ the available evidence is sparse and of lower quality. Policy-makers had little evidence to support the development of policies enacted during COVID-19 due to the rapidly evolving nature of COVID-19. In the evidence sources identified in our study, this led to variations across provinces. This variation may have also reflected the unique circumstances of each province (eg, local COVID-19 epidemiology and broader COVID-19 response policies, extent of surgical disruption, resource capacity). There is currently no definitive evidence to suggest that any specific policy option in the evidence sources identified in this study yielded better or worse outcomes for patients, healthcare providers, or the health systems during COVID-19. Moreover, there is no evidence to suggest that policies enacted during COVID-19, in haste and using little evidence, were any better or worse at achieving their objectives than those developed with more time and evidence. There is a need for implementation research to understand how current interventions are being adopted, identify barriers and facilitators to adoption, and examine how implementation methods may shape broader health system goals (eg, quality of care and equity).^51^ Such research will generate the evidence needed to achieve sustainable and scalable interventions that maximize benefits for patients, providers, and the health system.

 Moving forward, as the investment of resources into decreasing surgical wait times and the backlog of people waiting for surgery beyond COVID-19 increases it is critical that surgical care providers, healthcare administrators and health systems researchers work collaboratively to establish strategies and policies that are innovative and address more than access to surgical care. Patients whose surgeries were delayed during COVID-19 are likely to experience deteriorating health and poorer surgical outcomes,^11,52^ mental health issues including stress, anxiety and depression,^7,8,53,54^ and socioeconomic consequences resulting from lost productivity while waiting for surgery.^55^ Despite the far-reaching effects of surgical delays, the policies identified in the present study, and many others globally, focus almost exclusively on interventions to increase the capacity to complete more surgeries and do not appear to provide dedicated support to patients who are either waiting for surgery or have been adversely impacted by surgical delays. As governments commit resources towards surgical recovery interventions, it is important to also consider the overall well-being of patients waiting for surgery as wait times for surgery are likely to continue well beyond the context of COVID-19. In this regard, active collaboration with patients and patient advocates may help design low-cost, high-impact interventions tailored toward patients who are waiting to have surgeries.

 While our study has several strengths, including the use of evidence sources obtained from all Canadian jurisdictions and rigorous methods, there are also some limitations that need to be considered when interpreting our findings. We have made substantial efforts to retrieve policy documents from all provinces and territories (comprehensive search of online resources, received documents from team members who are surgical leaders, and interviewed leaders in provinces with sparse formal policies) to describe how surgical care was reorganized across Canada during COVID-19. However, our findings should be interpreted with the understanding that there may be a small number of internally circulated policies that we could not retrieve (as surgical care leaders in some provinces did not respond to our requests for documents). Similarly, three Canadian territories reported a paucity of evidence sources that described surgical care during COVID-19; however, after interviewing leaders from some of these provinces we believe it likely that these provinces relied on documents and policies from larger provinces and had fewer formal policy documents of their own. Also, because we have attempted to present information concisely, we may have omitted some specific details of provincial policies. To address this issue, we have included titles and links (for publicly available documents only) to the included policy documents in Table S1.

## Conclusion

 All Canadian provinces and territories had policies for reorganizing surgical care during COVID-19. Policies were responsive to changes in COVID-19 transmission dynamics and reflected provincial healthcare capacity and emerging evidence. Surgical recovery plans are currently being implemented across Canada and globally. Thus, there is need for collaborative research and evaluation to support current efforts towards improved access and quality of care for patients waiting for surgery.

## Acknowledgements

 We would like to acknowledge Drs. Liane Feldman and Ivar Mendez for their help in obtaining evidence sources.

## Ethical issues

 Ethical approval to conduct this study was obtained from the University of Calgary Conjoint Health Research Ethics Board (CHREB; ID: REB20-0753). Informed consent was obtained from healthcare leaders prior to beginning the interviews. All interview data was anonymized, stored on a secure server, and accessed by only approved members of the research team.

## Competing interests

 MB is on the Data and Safety Monitoring Board for a US randomized controlled trial on Enhanced Recovery After Surgery Society (ERAS) in pediatric patients, is the Secretary of the ERAS Society.

## Funding

 This work was supported by a grant from the Canadian Institutes for Health Research (Grant # 179887) to Khara M. Sauro. The funders had no input on study design; data collection, analysis, and interpretation.

## Supplementary files


Supplementary file 1. Data Abstraction Form.

Supplementary file 2 contains Table S1.

Supplementary file 3 contains Table S2.

Supplementary file 4 contains Table S3.

Supplementary file 5 contains Table S4.
