# Supplementary material for: Delivery and Prioritization of Surgical Care in Canada During COVID-19: An Environmental Scan
Source: Int J Health Policy Manag. 2023 Dec 10;12:8007. doi: 10.34172/ijhpm.2023.8007 (PMC10843432; doi:10.34172/ijhpm.2023.8007)
Supplement: Supplementary file 1 — Data Abstraction Form. [file ijhpm-12-8007-s001.pdf]

Article title: Delivery and Prioritization of Surgical Care in Canada During COVID-19: An Environmental Scan

Journal name: International Journal of Health Policy and Management (IJHPM)

Authors' information: Seremi Ibadin[1], Mary Brindle[2,3], Tracy Wasylak[3], Jill Robert[4], Stacey Litvinchuk[3], Khara M. Sauro[1,2,5]*

[1]Department of Community Health Sciences, Cumming School of Medicine, University of Calgary, Calgary, AB, Canada.

[2]Department of Surgery, Cumming School of Medicine, University of Calgary, Calgary, AB, Canada.

[3]Surgery Strategic Clinical Networks, Alberta Health Services, Calgary, AB, Canada.

[4]Surgery and Bone & Joint Health Strategic Clinical Networks, Alberta Health Services, Calgary, AB, Canada.

[5]Department of Oncology and Arnie Charbonneau Cancer Institute, Cumming School of Medicine, University of Calgary, Calgary, AB, Canada.

***Correspondence to:** Khara M. Sauro; Email: kmsauro@ucalgary.ca

Citation: Ibadin S, Brindle M, Wasylak T, Robert J, Litvinchuk S, Sauro KM. Delivery and prioritization of surgical care in Canada during COVID-19: an environmental scan. Int J Health Policy Manag. 2023;12:8007. doi:10.34172/ijhpm.2023.8007

Supplementary file 1. Data Abstraction Form

**Variable (Definition)**	**Data Extracted**
**Province**	
**Health Region**	
**City/Community**	
**Institution name**<br>(Including Department)	
**Document Author (s)**<br>(Please state the author(s) of the document)	
**Document title**<br>(Exactly as documented)	
**Source**<br>(Where was the document obtained?)	Select:<br>Publicly available webpage [ ]<br>Relevant Department/Institution [ ]<br>Other (specify) ---------
**Link**	
**Page(s) if applicable**	
**Document Type**	Select:

	Policy [ ]<br>Directive [ ]<br>Guideline [ ]<br>Memo [ ]<br>News Release/Update [ ]<br>Other ----------
**Document Scope**<br>(What jurisdiction did the document pertain to?)	Select:<br>Provincial [ ]<br>Health Authority [ ]<br>Institution [ ]<br>Department [ ]<br>Clinical Area [ ]<br>Other (specify) ---------
**Document Date**<br>(DD-MMM-YYYY)	
**Document Updated Date**<br>(DD-MMM-YYYY)	
**Document Effective Date**<br>(DD-MMM-YYYY)	
**Target Audience Category**<br>(Who was the document intended to provide guidance to?)	Select:<br>Provincial policy maker [ ]<br>Hospital policy maker [ ]<br>Department policy maker [ ]<br>Healthcare provider [ ]<br>Patients [ ]<br>Public [ ]<br>Other (specify) ---------
**Document Target Audience**<br>(Please state who the document was intended for exactly as documented)	Free text
**Policy Problem**<br>(Please state exactly as documented)	Free text
**Policy Goal/Objective**<br>(Please state exactly as documented)	Free text
**Policy Goal/Objective (category)**	Select:<br>Postpone non-urgent surgeries [ ]<br>Resume non-urgent surgeries [ ]<br>Triaging and prioritization of surgeries [ ]<br>IPC/safety measures [ ]<br>Reduce surgical backlog [ ]<br><br>Other (specify)----------
**Policy Content**<br>(Please abstract all relevant contents of the document)	Free text

**Relevant Quotes**	
**Additional notes**<br>(Include other notes related to document as relevant e.g., reference to related documents or information to aid data analysis)	
Does the document describe measures to increase the capacity of the health system to cope with surgical backlogs?	Yes [ ]<br>No [ ]
If yes, what capacity domains are described	Infrastructure e.g., build, renovate, expand operating rooms etc. [ ]<br>Staffing e.g., training, recruitment [ ]<br>Technology e.g., virtual care/teleconferencing [ ]<br>Administrative e.g., patient referral, patient flow [ ]<br>Outsourcing e.g., private or out-of-province facilities [ ]<br>Other (specify) ----------
Does the document describe additional funding or budgetary allocation to cope with surgical backlogs?	Yes [ ]<br>No [ ]
If yes, estimated funds or budgetary allocation	
Does the document include any measures of performance of strategies?	Yes [ ]<br>No [ ]
If yes, please document measured performance exactly as contained in the document (including dates)	
