# Supplementary material for: Delivery and Prioritization of Surgical Care in Canada During COVID-19: An Environmental Scan
Source: Int J Health Policy Manag. 2023 Dec 10;12:8007. doi: 10.34172/ijhpm.2023.8007 (PMC10843432; doi:10.34172/ijhpm.2023.8007)
Supplement: Supplementary file 2 — contains Table S1. [file ijhpm-12-8007-s002.pdf]

Article title: Delivery and Prioritization of Surgical Care in Canada During COVID-19: An Environmental Scan

Journal name: International Journal of Health Policy and Management (IJHPM)

Authors' information: Seremi Ibadin[1], Mary Brindle[2,3], Tracy Wasylak[3], Jill Robert[4], Stacey Litvinchuk[3], Khara M. Sauro[1,2,5]*

[1]Department of Community Health Sciences, Cumming School of Medicine, University of Calgary, Calgary, AB, Canada.

[2]Department of Surgery, Cumming School of Medicine, University of Calgary, Calgary, AB, Canada.

[3]Surgery Strategic Clinical Networks, Alberta Health Services, Calgary, AB, Canada.

[4]Surgery and Bone & Joint Health Strategic Clinical Networks, Alberta Health Services, Calgary, AB, Canada.

[5]Department of Oncology and Arnie Charbonneau Cancer Institute, Cumming School of Medicine, University of Calgary, Calgary, AB, Canada.

***Correspondence to:** Khara M. Sauro; Email: kmsauro@ucalgary.ca

Citation: Ibadin S, Brindle M, Wasylak T, Robert J, Litvinchuk S, Sauro KM. Delivery and prioritization of surgical care in Canada during COVID-19: an environmental scan. Int J Health Policy Manag. 2023;12:8007. doi:10.34172/ijhpm.2023.8007

Supplementary file 2

Table S1: Characteristics of Included Evidence Sources

S/N	Province/ Territory/ Federal	Institution	Document Author	Document Title	Document Type	Source	Document Date	Web Link
1.	AB	Alberta Health Services	Alberta Health Services	IPC Recommendations for suspected or confirmed COVID-19 Patients Requiring Surgery	Guideline	Publicly available webpage	18-Apr-20	https://www.albertahealthservices.ca/assets/healthinfo/ipc/hi-ipc-suspect-conf-emergency-urgent-surgery.pdf
2.	AB	Alberta Health Services	Clinical Department Head, Surgery	Relaunch Plan for Surgery - Information	Memo	Relevant department	1-May-20	NA

3.	AB	Alberta Health Services	Alberta Health Services (Alberta Surgical Initiative)	Resumption of Surgery in Alberta: A Staged Approach to COVID-19 Surgical Recovery	Plan	Relevant department	5-Jun-20	NA
4.	AB	Alberta Health Services	Alberta Health Services	Annual Report 2019 - 2020 (Fiscal Year April 1, 2019 to March 31, 2020)	Report	Publicly available webpage	24-Jun-20	https://www.albertahealthservices.ca/assets/about/publications/2019-20-annual-report-web-version.pdf
5.	AB	Alberta Health Services	Alberta health Services	AHS Performance Review Proposed Implementation Plan	Plan	Publicly available webpage	13-Aug-20	https://open.alberta.ca/dataset/e07fb93b-806f-4d91-bceb-640ea4ba5473/resource/c4890bfa-bc7c-48e2-8c95-e34c9c15f1da/download/health-ahs-review-implementation-plan-2020-08.pdf
6.	AB	Alberta Government Ministry of Health	Alberta Government Ministry of Health	COVID-19 scheduled surgery backlog cut by 88 per cent	News Release	Publicly available webpage	11-Sep-20	https://www.alberta.ca/release.cfm?xID=73205E921E67C-EAAC-9C7F-6E13A6B30D2AEB36
7.	AB	Alberta Health Services	Alberta Health Services	Point-of-care risk assessment for Surgery during COVID-19	Guideline	Publicly available webpage	8-Dec-20	https://www.albertahealthservices.ca/assets/healthinfo/ipc/hi-ipc-covid-19-or-algorithm.pdf
8.	AB	Alberta Health Services	Alberta Health Services	IPC PPE table for surgical suites	Guideline	Publicly available webpage	17-Dec-20	https://www.albertahealthservices.ca/assets/healthinfo/ipc/hi-ipc-ppe-tbl-surg-covid-19.pdf

9.	AB	Alberta Health Services	Alberta Health Services	Surgical Services: Pandemic Planning & Mitigation Strategies - Framework for Surgical Services Slowdowns	Guideline	Relevant department	18-Dec-20	NA
10.	AB	Government of Alberta	KPMG	Review of Alberta's COVID-19 Pandemic Response: March 1 to October 12, 2020	Report	Publicly available webpage	1-Jan-21	https://www.alberta.ca/assets/documents/health-alberta-covid-19-pandemic-response-review-final-report.pdf
11.	AB	Alberta Government	Alberta Government	Budget 2021: More funding to reduce surgical wait times	News Release	Publicly available webpage	5-Mar-21	https://www.alberta.ca/release.cfm?xID=77663291345CB-BE69-E9A8-171C5F17479D2BDC
12.	AB	College of Physicians and Surgeons of Alberta	College of Physicians and Surgeons of Alberta	From AHS: Surgical COVID-19 Wave 2 Recovery Plan	News Release	Publicly available webpage	8-Apr-21	https://cpsa.ca/news/from-ahs-surgical-covid-19-wave-2-recovery-plan/
13.	AB	Alberta Health Services	Alberta Health Services	AHS postpones some non-urgent surgeries to create additional hospital capacity	News Release	Publicly available webpage	27-Aug-21	https://www.albertahealthservices.ca/news/Page16153.aspx
14.	AB	Alberta Health Services	Alberta Health Services	CMO SMOH Notice for AHS Medical Staff	Memo	Publicly available webpage	27-Aug-21	https://www.albertahealthservices.ca/assets/info/ppih/if-ppih-covid-19-cmo-update-2021-08-27.pdf
15.	AB	Alberta Health Services	Alberta Health Services	AHS postpones scheduled surgeries due to COVID-19	News Release	Publicly available webpage	3-Sep-21	https://www.albertahealthservices.ca/news/Page16174.aspx
16.	AB	Alberta Health Services	Alberta Health Services	AHS Continues to schedule prioritized cancer surgeries	News Release	Publicly available webpage	9-Sep-21	https://www.albertahealthservices.ca/news/Page16186.aspx

17.	AB	Alberta Health Services	Alberta Health Services	Untitled_To ensure we are working as a consolidated system, all zones are expected to implement the following	Guideline	Relevant department	15-Sep-21	NA
18.	AB	Alberta Health Services	Alberta Health Services (Vice President & Chief Operating Officer, Clinical Operations & Vice President & Medical Director, Clinical Operations	Immediate Activation of Highest Level Surge Response Plans	Memo	Relevant department	15-Sep-21	NA
19.	AB	Alberta Health Services	Alberta Health Services	AHS Facilities Temporary Bed/Space Reduction: Surgical reduction by zone due to COVID-19	News Release	Publicly available webpage	20-Sep-21	https://www.albertahealthservices.ca/br/Page17709.aspx
20.	AB	Alberta Health Services	Alberta Health Services (Vice President & Chief Operating Officer, Clinical Operations & Vice President & Medical Director, Clinical Operations, Provincial Surgery Operations committee Co-Chairs, Alberta Surgical Initiative Lead	Immediate Activation of Highest Level Surge Response Plans: Surgical Guidelines Updated	Memo	Relevant department	22-Sep-21	

21.	AB	Alberta Health Services	Alberta Health Services (Vice President & Chief Operating Officer, Clinical Operations & Vice President & Medical Director, Clinical Operations, Provincial Surgery Operations committee Co-Chairs, Alberta Surgical Initiative Lead	Immediate Activation of Highest Level Surge Response Plans: Surgical Guidelines Updated	Memo	Relevant department	24-Sep-21	NA
22.	AB	Alberta Health Services	Alberta Health Services	Cancer Surgery Pandemic Protocol	Memo	Relevant department	28-Sep-21	NA
23.	AB	Alberta Health Services	Alberta Health Services (Strategic Clinical Networks)	Post COVID-19 Playbook: Managing Impact and Maximizing Resiliency of Alberta's Health System_Draft	Plan	Relevant department	1-Oct-21	NA
24.	AB	Alberta Health Services	Alberta Health Services (Alberta Surgical Initiative)	Alberta Pandemic Surgery Resumption Framework: A Framework to Support Graduated Approach to Surgical Service Reinstatement	Plan	Relevant department	13-Oct-21	NA
25.	AB	Alberta Health Services	Alberta Health Services	2021-22 Bi-Annual Performance Report: Measuring Progress on Year 2 of the AHS 2020-2022 Health Plan April 1, 2021 - Spetember 30, 2021	Report	Publicly available webpage	14-Dec-21	https://www.albertahealthservices.ca/assets/about/publications/ahs-pub-pr-2021-22-q2.pdf

26.	AB	Alberta Health Services	Alberta Health Services (Surgery Strategic Clinical Network)	Guidance for surgery after COVID-19 infection: Timing of surgery following recovery from COVID-19 TO reduce risk of postoperative mortality	Guideline	Publicly available webpage	20-Jan-22	https://www.albertahealthservices.ca/assets/info/ppih/if-ppih-covid-19-guidance-surgery-after-covid-19.pdf
27.	AB	Government of Alberta	Government of Alberta	Budget 2022 Fiscal Plan: Moving Forward	Plan	Publicly available webpage	24-Feb-22	https://open.alberta.ca/publications/budget-2022
28.	AB	Government of Alberta	Government of Alberta	Budget 2022: Increasing health-care capacity, surgeries	Plan	Publicly available webpage	25-Feb-22	https://corealberta.ca/news/budget-2022-increasing-health-care-capacity-surgeries
29.	AB	Alberta Health Services	Alberta Health Services	Alberta Provincial Surgery Recovery Plan: Application of the Alberta Surgical Initiative as the Framework for Pandemic Recovery and Surgery Wait Time Reduction	Plan	Relevant department	11-Apr-22	NA
30.	AB	Alberta Health Services	Alberta Health Services (Interim AHS President and CEO & SMOH)	Messages from Mauro Chies & Dr. Laura McDougall - AHS Update: Surgical wait list reduced, COVID-19 hospitalizations and ICU admission on rise, Health Link sets new record for annual callers	Memo	Publicly available webpage	14-Apr-22	

31.	AB	Alberta Health Services	Alberta Health Services (Interim AHS President and CEO & SMOH)	Messages from Mauro Chies & Dr. Laura McDougall - AHS Update: Expanding Access to Surgery in Central and South Zones…	Memo	Publicly available webpage	9-Sep-22	https://www.albertahealthservices.ca/assets/info/ppih/if-ppih-covid-19-ceo-message-2022-04-14.pdf
32.	BC	Northern Health	Northern Health	Non-urgent scheduled surgeries postponed	News Release	Publicly available webpage	17-Mar-20	https://stories.northernhealth.ca/news/non-urgent-scheduled-surgeries-postponed
33.	BC	Provincial Health Services Authority	Provincial Health Services Authority	Provincial Cancer Clinical Management Guidelines in Pandemic Situation (COVID-19) Current Version as of April 14, 2020	Guideline	Publicly available webpage	14-Apr-20	http://www.bccancer.bc.ca/health-professionals-site/Documents/Provincial Cancer Therapy Clinical Management Guidelines in Pandemic situation (COVID-19)_April 14 2020.pdf
34.	BC	BC Ministry of Health	BC Ministry of Health	A Commitment to Surgical Renewal in B.C.	Plan	Publicly available webpage	7-May-20	https://www2.gov.bc.ca/assets/gov/health/conducting-health-research/surgical-renewal-plan.pdf
35.	BC	Fraser Health	Fraser Health	Virtual pre-admission for surgery to keep you safe at home	News Release	Publicly available webpage	11-May-20	https://www.fraserhealth.ca/news/2020/May/virtual-pre-admission-for-surgery-to-keep-you-safe-at-home -.Yrmo2uzMKqA

36.	BC	Island Health	Island Health	Island Health begins implementing surgical renewal plan	News Release	Publicly available webpage	12-May-20	https://www.islandhealth.ca/news/news-releases/island-health-begins-implementing-surgical-renewal-plan
37.	BC	Vancouver Coastal Health	Vancouver Coastal Health	Daily update: Keeping patients safe as scheduled elective surgeries resume	News Release	Publicly available webpage	15-May-20	http://www.vch.ca/Documents/COVID-19-Daily-Update-May-15.pdf
38.	BC	Fraser Health	Fraser Health	Fraser Health provides update on elective surgeries	News Release	Publicly available webpage	29-May-20	https://www.fraserhealth.ca/news/2020/May/fraser-health-provides-update-on-elective-surgeries - .YrnY0OzMK3I
39.	BC	Provincial Health Services Authority	Provincial Health Services Authority	Provincial Health Service Authority 2020//21 - 2022/23 Service Plan	Plan	Publicly available webpage	17-Sep-20	http://www.phsa.ca/about-site/Documents/2020-21-2022-23-Service-Plan.pdf
40.	BC	BC Ministry of Health	BC Ministry of Health	BC continues to advance surgical renewal achievement	News Release	Publicly available webpage	19-Mar-21	https://news.gov.bc.ca/releases/2021HLTH0055-000513
41.	BC	BC Government	BC Government	Province strengthens COVID-19 measures for safer holiday season	News Release	Publicly available webpage	21-Dec-21	https://news.gov.bc.ca/releases/2021HLTH0234-002431
42.	BC	BC Ministry of Health	BC Ministry of Health	Options for Operating Room Configuration and Use When a Patient with Suspected or Confirmed COVID-19 Requires Emergent Surgery	Guideline	Publicly available webpage	12-Jan-22	http://www.bccdc.ca/Health-Professionals-Site/Documents/COVID19_ORConfigUseEmergentSurgery.pdf

43.	BC	BC Ministry of Health	BC Ministry of Health	Year 2 of surgical renewal delivers most surgeries ever completed in a year in B.C.	News Release	Publicly available webpage	4-May-22	https://news.gov.bc.ca/releases/2022HLTH0134-000708
44.	BC	BC Ministry of Health	BC Ministry of Health	Infection Prevention and Control (IPC) Protocol for Adult Surgical Procedures During the Covid-19 Pandemic	Guideline	Publicly available webpage	11-May-22	http://www.bccdc.ca/Health-Professionals-Site/Documents/COVID19_IPCProtocolSurgicalProceduresAdult.pdf
45.	BC	BC Ministry of Health	BC Ministry of Health	Infection Prevention and Control (IPC) Protocol for Paediatric Surgical Procedures During the Covid-19 Pandemic	Guideline	Publicly available webpage	Undated	http://www.bccdc.ca/Health-Professionals-Site/Documents/COVID19_IPCProtocolSurgicalProceduresPediatric.pdf
46.	BC	BC Ministry of Health	BC Ministry of Health	COVID-19 and Adult Surgeries: How We Are Keeping You Safe	Informational /Handout	Publicly available webpage	Undated	http://www.bccdc.ca/Health-Professionals-Site/Documents/COVID-19_Adult_Surgical_Patient_Handout.pdf
47.	BC	BC Ministry of Health	BC Ministry of Health	COVID-19 and Paediatric Procedure: How We Are Keeping Your Child Safe	Informational /Handout	Publicly available webpage	Undated	http://www.bccdc.ca/Health-Professionals-Site/Documents/COVID-19_Pediatric_Surgical_Patient_Handout.pdf
48.	MB	Manitoba Government	Manitoba Government	COVID-19 Bulletin #16	News Release	Publicly available webpage	18-Mar-20	https://news.gov.mb.ca/news/index.html?item=46997
49.	MB	Manitoba Government	Manitoba Government	COVID-19 Bulletin #61	News Release	Publicly available webpage	24-Apr-20	https://news.gov.mb.ca/news/?archive=&item=47621

50.	MB	Manitoba Government	Manitoba Government	Province issues request to address surgery backlog	News Release	Publicly available webpage	2-Jul-20	https://news.gov.mb.ca/news/index.html?item=48549&posted=2020-07-02
51.	MB	Manitoba Government	Manitoba Government	Province awards contracts to address surgery backlog	News Release	Publicly available webpage	17-Aug-20	https://news.gov.mb.ca/news/index.html?item=49083&posted=2020-08-17
52.	MB	Manitoba Government	Manitoba Government	Southern Health-Sante Sud moves to critical (red) on RESTARTMB pandemic response system	News Release	Publicly available webpage	6-Nov-20	https://news.gov.mb.ca/news/index.html?item=49617&posted=2020-11-06
53.	MB	Manitoba Government	Manitoba Government	Restart MB Pandemic Response System	News Release	Publicly available webpage	7-Jan-21	https://www.gov.mb.ca/asset_library/en/restartmb/pandemic_response_system.pdf
54.	MB	Manitoba Government	Manitoba Government	Province adding surgical capacity to begin addressing surgery backlog	News Release	Publicly available webpage	8-Jan-21	https://news.gov.mb.ca/news/index.html?item=50202
55.	MB	Shared Health	Shared Health	COVID-19 Operating Room Risk Stratification for Surgical Patients	Guideline	Publicly available webpage	23-Feb-21	https://sharedhealthmb.ca/files/covid-19-risk-stratification-for-surgical-patients.pdf
56.	MB	Manitoba Government	Manitoba Government	Province commits $1.2 billion for COVID-19 Response in Budget 2021	News Release	Publicly available webpage	6-Apr-21	https://news.gov.mb.ca/news/index.html?item=51079&posted=2021-04-06
57.	MB	Manitoba Government	Manitoba Government	Province steps up preparedness for COVID-19 fourth wave, moves to restricted (orange) level on pandemic response system	News Release	Publicly available webpage	1-Oct-21	https://news.gov.mb.ca/news/index.html?item=52562

58.	MB	Manitoba Government	Manitoba Government	Task Force established to address diagnostic and surgical backlogs	News Release	Publicly available webpage	8-Dec-21	https://news.gov.mb.ca/news/index.html?item=52823
59.	MB	Shared Health	Shared Health	Surgical Prioritization Underway	News Release	Publicly available webpage	19-Dec-21	https://sharedhealthmb.ca/news/2021-12-19-surgical-prioritization/
60.	MB	Manitoba Government	Manitoba Government	Diagnostic and Surgical Recovery Task Force Delivering Improvements for Manitobans	News Release	Publicly available webpage	19-Jan-22	https://www.gov.mb.ca/dsrecovery/index.html
61.	MB	Manitoba Government	Manitoba Government	Manitoba provides update on the diagnostic and surgical recovery task force	News Release	Publicly available webpage	30-Mar-22	https://news.gov.mb.ca/news/?archive=&item=54024
62.	MB	Manitoba Government	Manitoba Government	Budget 2022 provides historic $7.2 billion investment to strengthen health care for all Manitobans	News Release	Publicly available webpage	12-Apr-22	https://news.gov.mb.ca/news/index.html?item=54179 - :~:text=Budget 2022 prioritizes the health,Minister Cameron Friesen announced today.
63.	MB	Shared Health	Shared Health	Provincial Guidance for COVID-19: A Return to Symptomatic Testing Infection Prevention and Control Risk and Response	Guideline	Publicly available webpage	15-Jun-22	https://sharedhealthmb.ca/files/covid-19-admission-preop-testing.pdf

64.	MB	Health Sciences Centre Foundation	Health Sciences Centre Foundation	Health Sciences Centre Foundation Launches "operation Excellence" - A $100 Million Capital Campaign to Develop Surgical and Diagnostic Capacity at HSC, Manitoba's Hospital	News Release	Publicly available webpage	22-Jun-22	https://www.hscfoundation.mb.ca/announcements/health-sciences-centre-foundation-launches-operation-excellence-a-100-million-capital-campaign-to-develop-surgical-and-diagnostic-capacity-at-hsc-manitobas-hospital/
65.	MB	Doctors Manitoba	Doctors Manitoba	Surgery & Diagnostic Backlog: New Estimates & Progress	Report	Publicly available webpage	28-Jun-22	https://doctorsmanitoba.ca/about-us/advocacy-policy/backlog
66.	MB	Manitoba Government	Manitoba Government	Manitoba Government connecting patients with faster hip and knee surgeries through partnerships	News Release	Publicly available webpage	24-Aug-22	https://news.gov.mb.ca/news/?archive=&item=56058
67.	MB	Manitoba Government	Manitoba Government	Manitoba Virtual Care Action Plan	Plan	Publicly available webpage	7-Oct-22	https://www.canada.ca/en/health-canada/corporate/transparency/health-agreements/bilateral-agreement-pan-canadian-virtual-care-priorities-covid-19/manitoba-action-plan.html - :~:text=VCOP provides service to patients,or following discharge from hospital.

68.	MB	Manitoba Government	Manitoba Government	Diagnostic and Surgical Recovery Task Force	Plan	Publicly available webpage	Undated	https://www.gov.mb.ca/dsrecovery/index.html
69.	NB	Government of New Brunswick	Office of the Premier	Revised/ State of emergency declared in response to COVID-19	News Release	Publicly available webpage	19-Mar-20	https://www2.gnb.ca/content/gnb/en/news/news_release.2020.03.0139.html
70.	NB	Horizon Health Network	Horizon Health Network Presdient and CEO	CEO Statement: Horizon is entering a new phase in its response to COVID-19	News Release	Publicly available webpage	8-May-20	https://horizonnb.ca/news-releases/ceo-statement-horizon-is-entering-a-new-phase-in-its-response-to-covid-19/
71.	NB	Government of New Brunswick	Office of the Premier	Province moves to Phase 2 (Orange) of recovery plan; no new cases of COVID-19	News Release	Publicly available webpage	8-May-20	https://www2.gnb.ca/content/gnb/en/news/news_release.2020.05.0261.html
72.	NB	New Brunswick Medical Society	President of New Brunswick Medical Society	Moving health care forward in New Brunswick during COVID-19	News Release	Publicly available webpage	23-May-20	https://www.fmnb.ca/moving-health-care-forward-in-new-brunswick-during-covid-19/
73.	NB	Horizon Health Network	Horizon Health Network	Services to continue at Horizon's The Moncton Hospital during COVID-19 outbreak	News Release	Publicly available webpage	8-Oct-20	https://horizonnb.ca/news-releases/services-to-continue-at-horizons-the-moncton-hospital-during-covid-19-outbreak/
74.	NB	Vitale Health Network	Vitale Health Network	Vitale Health Network adjusting to the transition to Orange level in the Edmundston region (Zone 4)	News Release	Publicly available webpage	11-Dec-20	https://www.vitalitenb.ca/en/news/vitalite-health-network-adjusting-transition-orange-level-edmundston-region-zone-4

75.	NB	Vitale Health Network	Vitale Health Network	COVID-19: All Vitale Health Network hospitals and facilities in red alert phase	News Release	Publicly available webpage	7-Oct-21	https://www.vitalitenb.ca/en/news/covid-19-all-vitalite-health-network-hospitals-and-facilities-red-alert-phase
76.	NB	Vitale Health Network	Vitale Health Network	COVID-19: Vitale Health Network adjusts to return to yellow phase in Zone 1 (Moncton region)	News Release	Publicly available webpage	23-Oct-21	https://www.vitalitenb.ca/en/news/covid-19-vitalite-health-network-adjusts-return-yellow-phase-zone-1-moncton-region
77.	NB	Horizon Health Network	Horizon Health Network	Status Report - COVID-19 Outbreaks and Hospital Operations	News Release	Publicly available webpage	21-Dec-21	https://horizonnb.ca/news-releases/status-report-covid-19-outbreaks-and-hospital-operations-4/
78.	NB	Vitale Health Network	Vitale Health Network	COVID-19: Alert Level Increase in All Network Facilities	News Release	Publicly available webpage	31-Dec-21	https://www.vitalitenb.ca/en/news/covid-19-alert-level-increase-all-network-facilities
79.	NB	Horizon Health Network	Horizon Health Network	Status Report - Horizon hospitals return to Red alert	News Release	Publicly available webpage	31-Dec-21	https://stancassidy.ca/home/media-centre/horizon-news/20211231_horizon_status_rapport_red_phase_protocols_nr.aspx
80.	NB	Government of New Brunswick	Government of New Brunswick	New testing and isolation measures to mitigate impacts of Omicron variant/school moving to home learning	News Release	Publicly available webpage	31-Dec-21	https://www2.gnb.ca/content/gnb/en/departments/education/news/news_release.2021.12.0944.html
81.	NB	Horizon Health Network	Horizon Health Network	Status Report - Hospital Operations	News Release	Publicly available webpage	7-Jan-22	https://horizonnb.ca/news-releases/status-report-hospital-operations/

82.	NB	Horizon Health Network	Horizon Health Network	Horizon resuming non-urgent surgeries, outpatient appointments	Report	Publicly available webpage	12-Feb-22	https://horizonnb.ca/news-releases/horizon-resuming-non-urgent-surgeries-outpatient-appointments/
83.	NB	Vitale Health Network	Vitale Health Network	Community Update - February 18, 2022: Situation Report	News Release	Publicly available webpage	18-Feb-22	https://www.vitalitenb.ca/en/news/community-update-february-18-2022
84.	NB	Vitale Health Network	Vitale Health Network	Community Update - April 1, 2022: Situation Report	News Release	Publicly available webpage	1-Apr-22	https://www.vitalitenb.ca/en/news/community-update-april-1-2022
85.	NB	Department of Health	Department of Health	Annual Report 2020 - 2021	Report	Publicly available webpage	Undated	https://www2.gnb.ca/content/dam/gnb/Departments/h-s/pdf/en/Publications/AnnualReport_2020-2021.pdf
86.	NB	Department of Health	Department of Health	Stabilizing Health Care: An Urgent Call to Action	Plan	Publicly available webpage	Undated	https://www2.gnb.ca/content/dam/gnb/Departments/h-s/pdf/Stabilizing-health-care.pdf
87.	NB	Robert Wood Johnson University Hospital	Robert Wood Johnson University Hospital	Safe surgery during COVID-19 at RWJUH New Brunswick: Keeping everyone safe	Informational /Handout	Publicly available webpage	Undated	https://www.rwjbh.org/rwj-university-hospital-new-brunswick/patients-visitors/temporary-changes-to-services-and-visitation-pol/safe-surgery-during-covid-19/
88.	NB	Vitale Health	Vitale Health	Preparing for surgery (Orange level)	Informational /Handout	Publicly available webpage	Undated	https://www.vitalitenb.ca/en/covid-

								19/preparing-for-surgery
89.	NL	Western Health	Western Health	Western Health to resume some services	News Release	Publicly available webpage	13-Mar-20	https://www.westernhealth.nl.ca/news/841/603/Western-Health-to-Resume-Some-Services/
90.	NL	Western Health	Western Health	Western Health imposes restriction	News Release	Publicly available webpage	15-Mar-20	https://westernhealth.nl.ca/news/830/603/Western-Health-Imposes-Restrictions/
91.	NL	Central Health	Central Health	Central Health to reduce elective and non-urgent services and procedures around the region	News Release	Publicly available webpage	19-Mar-20	https://www.centralhealth.nl.ca/post/central-health-to-reduce-elective-and-non-urgent-services-and-procedures-around-the-region - :~:text=Central Health is advising the,Chief Medical Officer of Health.&text=Only urgent%2Femergent surgeries will take place.&text=All other elective surgeries will be rebooked.
92.	NL	Western Health	Western Health	Western Health Increasing Services During Alert Level 3	News Release	Publicly available webpage	10-Jun-20	https://westernhealth.nl.ca/news/845/603/Western-Health-Increasing-Services-During-Alert-Level-3/

93.	NL	Central Health	Central Health	Central Health to reduce elective and non-urgent services and procedures around the region	News Release	Publicly available webpage	15-Feb-21	https://www.centralhealth.nl.ca/post/central-health-to-reduce-elective-and-non-urgent-services-and-procedures-around-the-region-1
94.	NL	Central Health	Central Health	Central health to reduce elective and non-urgent services and procedures under Alert Level 4	News Release	Publicly available webpage	24-May-21	https://www.centralhealth.nl.ca/post/central-health-to-reduce-elective-and-non-urgent-services-and-procedures-under-alert-level-4
95.	NL	Central Health	Central Health	Annual Performance Report 2020 - 2021	Report	Publicly available webpage	29-Jun-21	https://www.assembly.nl.ca/business/electronicdocuments/CentralHealthAnnualReport2020-21.pdf
96.	NL	Newfoundland and Labrador Medical Association	Director of Communications & Public Affairs	Doctors sound alarm over cancelled surgeries and delayed cancer care	News Release	Publicly available webpage	11-Mar-22	https://findadoctornl.ca/site/uploads/2022/03/2022.03.11-News-Release-Doctors-sound-alarm-over-cancelled-surgeries-and-delayed-cancer-care-FINAL-1.pdf
97.	NL	Government of Newfoundland and Labrador	Government of Newfoundland and Labrador	Surgical Task Force Members Announced	News Release	Publicly available webpage	16-Jun-22	https://www.gov.nl.ca/releases/2022/exec/0616n01/

98.	NL	Eastern Health	Eastern Health	Strategic Health Plan 2020 - 2023	Plan	Publicly available webpage	Undated	https://www.easternhealth.ca/wp-content/uploads/2021/05/Eastern-Health_Strategic-Plan_2020-23.pdf
99.	NS	Nova Scotia Health Authority	Nova Scotia Health Authority	Many NSHA services reduced or suspended as part of COVID-19 response	News Release	Publicly available webpage	17-Mar-20	https://www.nshealth.ca/news/many-nsha-services-reduced-or-suspended-part-covid-19-response
100.	NS	Nova Scotia Health Authority	Nova Soctia Health Authority	NSHA Perioperative and Interventional Radiology Services During COVID-19 Pandemic: Recommendations for Triage of Urgent, Benign and Cancer Surgery	Guideline	Publicly available webpage	20-Mar-20	https://www.csnscn.ca/images/Perioperative_and_IR_Services_During_COVID.3.20.4pm_1.pdf
101.	NS	Nova Scotia Health Authority	Nova Scotia Health Authority	Surgical services at Aberdeen Hospital to reopen April 11	News Release	Publicly available webpage	9-Apr-20	https://nshealth.ca/news/surgical-services-aberdeen-hospital-reopen-april-11
102.	NS	Nova Scotia Health Authority	Nova Scotia Health Authority	NSHA addresses "community clusters" document on social media	News Release	Publicly available webpage	29-Apr-20	https://www.nshealth.ca/news/nsha-addresses-community-clusters-document-social-media - :~:text=NSHA addresses "community clusters" document on social media,-Wednesday, April 29&text=We are aware of Nova,or spread of COVID-19.

103.	NS	Nova Scotia Health Authority	Nova Scotia Health Authority	Preparing for surgery: what to expect	Informational /Handout	Publicly available webpage	1-Jun-20	https://www.nshealth.ca/files/preparing-surgery-what-expect
104.	NS	Nova Scotia Health Authority	Nova Scotia Health Authority	COVID-19 service reintroduction update #5	Report	Publicly available webpage	22-Sep-20	https://www.nshealth.ca/files/covid-service-reintroduction-update-5-sept-22pdf
105.	NS	Nova Scotia Health	Nova Scotia Health	Hip and Knee Action Plan Update_October 2020	Report	Publicly available webpage	1-Oct-20	https://www.nshealth.ca/sites/nshealth.ca/files/hip-and-knee-action-plan-update_-_oct20_2020.pdf
106.	NS	Nova Scotia Health Authority	Krista Woods	COVID-19 requires teams to rethink how they deliver surgical care	News Release	Publicly available webpage	18-Dec-20	https://www.nshealth.ca/news/covid-19-requires-teams-rethink-how-they-deliver-surgical-care
107.	NS	Nova Scotia Government	Minister, Finance and Treasury Board	Budget 2021-22_A fair and prosperous path to balance_budget address	Plan	Publicly available webpage	25-Mar-21	https://beta.novascotia.ca/sites/default/files/documents/7-2634/address-budget-2021-2022-en.pdf
108.	NS	Nova Scotia Health Authority	Nova Scotia Health Authority	Temporary reduction of surgeries at QEII, Dartmouth General Hospitals	News Release	Publicly available webpage	23-Apr-21	https://www.nshealth.ca/news/temporary-reduction-surgeries-qeii-dartmouth-general-hospitals
109.	NS	Nova Scotia Health Authority	Nova Scotia Health Authority	Surgeries postponed at Glace Bay, Northside General hospitals for Monday, Tuesday	News Release	Publicly available webpage	25-Apr-21	https://www.nshealth.ca/news/surgeries-postponed-glace-bay-northside-general-hospitals-monday-tuesday

110.	NS	Nova Scotia Health Authority	Nova Scotia Health Authority	Temporary service reductions throughout Nova Scotia	News Release	Publicly available webpage	30-Apr-21	https://www.nshealth.ca/news/temporary-service-reductions-throughout-nova-scotia
111.	NS	Nova Scotia Health Authority	Nova Scotia Health Authority	Nova Scotia Health resuming services temporarily reduced during third wave	News Release	Publicly available webpage	14-Jun-21	https://www.nshealth.ca/news/nova-scotia-health-resuming-services-temporarily-reduced-during-third-wave
112.	NS	Nova Scotia Health Authority	Nova Scotia Health Authority	Service reductions continue due to increased emergency visits and admissions, staffing challenges	News Release	Publicly available webpage	19-Nov-21	https://www.nshealth.ca/news/correction-service-reductions-continue-due-increased-emergency-visits-and-admissions-staffing
113.	NS	Nova Scotia Health Authority	Nova Scotia Health Authority	QEII will postpone some non-urgent surgeries beginning Monday	News Release	Publicly available webpage	17-Dec-21	https://nshealth.ca/news/qeii-will-postpone-some-non-urgent-surgeries-beginning-monday
114.	NS	Goverenment of Nova Scotia	Goverenment of Nova Scotia	Budget 2022 to 2023: Solutions for Health Care, Solutions for Nova Scotians	Plan	Publicly available webpage	29-Mar-22	https://beta.novascotia.ca/sites/default/files/documents/6-3059/ftb-bfi-044-en-budget-2022-2023.pdf
115.	NS	Nova Scotia Health Authority	Perioperative (Surgical) Services Network	Surgical Access and Quality Improvement Strategy	Plan	Publicly available webpage	19-May-22	https://www.engage4health.ca/perioperative-network

116.	NS	Nova Scotia Health Authority	Nova Scotia Health Authority	Decision and Management Protocol for Surgical Procedures Requiring Anesthesia during COVID-19	Guideline	Publicly available webpage	23-May-22	https://policy.nshealth.ca/Site_Published/covid19/document_render.aspx?documentRender.IdType=6&documentRender.GenericField=&documentRender.Id=83387
117.	NS	Nova Scotia Health Authority	Krista Woods	Same-day joint replacement helps support access to surgeries during COVID-19	News Release	Publicly available webpage	16-Oct-22	https://www.nshealth.ca/news/same-day-joint-replacements-help-support-access-surgeries-during-covid-19
118.	NS	Nova Scotia Health Authority	Nova Scotia Health Authority	Action for Health: A Strategic Plan 2022 - 2026	Plan	Publicly available webpage	Undated	https://novascotia.ca/actionforhealth/docs/action-for-health-strategic-plan-for-nova-scotia.pdf
119.	NS	Nova Scotia Health	Nova Scotia Health	Annual Report 2020	Report	Publicly available webpage	Undated	https://www.nshealth.ca/sites/nshealth.ca/files/nsha_ri_annual_report_2020_draft_july_final_pages.pdf
120.	NT	Northwest Territories Health and Social Services Authority	Northwest Territories Health and Social Services Authority	Public Notice - NTHSSA - Service Changes - March 19, 2020	News Release	Publicly available webpage	19-Mar-20	https://www.nthssa.ca/en/newsroom/public-notice-nthssa-service-changes-march-19-2020
121.	NT	Northwest Territories Health and Social Services Authority	Northwest Territories Health and Social Services Authority	COVID-19 Response Team's (ACRT): Pandemic Response Plan for Health Services	News Release	Publicly available webpage	1-Sep-20	https://www.nthssa.ca/sites/nthssa/files/resources/authoritiescovid19pandemicresponseplan_final_2020.09_web_0.pdf

122.	NT	Northwest Territories Health and Social Services Authority	Northwest Territories Health and Social Services Authority	Public Notice: Service Level Update for Stanton and Yellowknife Region	News Release	Publicly available webpage	8-Sep-21	https://www.nthssa.ca/en/newsroom/public-notice-service-level-update-stanton-and-yellowknife-region
123.	NT	Northwest Territories Health and Social Services Authority	Northwest Territories Health and Social Services Authority	Update: Service Reductions to Primary Care and Stanton Hospital in Yellow Knife	News Release	Publicly available webpage	12-Jan-22	https://www.nthssa.ca/en/newsroom/updated-service-reductions-primary-care-and-stanton-hospital-yellowknife
124.	NT	Northwest Territories Health and Social Services Authority	Northwest Territories Health and Social Services Authority	COVID Testing	Informational /Handout	Publicly available webpage	Undated	https://www.nthssa.ca/en/covid-testing - :~:text=Tests are available in your community%3A&text=COVID-19 testing to inform,ordered by a healthcare provider.
125.	NU	Government of Nunavut	Government of Nunavut	COVID-19 GN Update - April 15 2021	News Release	Publicly available webpage	15-Apr-21	https://www.gov.nu.ca/sites/default/files/gn_covid-19_update_april_15_2021_-_eng.pdf
126.	NU	Government of Nunavut	Government of Nunavut	COVID-19 GN Update - December 29 2021	News Release	Publicly available webpage	29-Dec-21	https://www.gov.nu.ca/health/news/covid-19-department-health-services-update
127.	ON	Ontario Health	Cancer Care Ontario	Pandemic Planning Clinical Guideline for Patients with Cancer	Guideline	Publicly available webpage	10-Mar-20	https://www.cancercareontario.ca/sites/ccocancercare/files/guidelines/full/PandemicPlanningClinicalGuidelines-Cancer.pdf

128.	ON	Ontario Ministry of Health	Deputy Minister, Chief Medical Officer of Health, President and CEO Ontario Health	Ramping Down Elective Surgeries and Other Non-Emergent Activities	Memo	Publicly available webpage	15-Mar-20	https://www.health.gov.on.ca/en/pro/programs/publichealth/coronavirus/docs/memos/DM_OH_CMOH_memo_COVID19_elective_surgery_March_15_2020.pdf
129.	ON	Ontario Health	Ontario Health	Clinical Triage Protocol for Major Surge in COVID Pandemic	Memo	Publicly available webpage	28-Mar-20	https://med.uottawa.ca/pathology/sites/med.uottawa.ca.pathology/files/clinical_triage_protocol_for_major_surge_in_covid_pandemic_-_march_28_20205.pdf
130.	ON	Ontario Health	Cancer Care Ontario	COVID-19 Supplemental Clinical Guidance for Patients with Cancer	Guideline	Publicly available webpage	29-Mar-20	https://www.ontariohealth.ca/sites/ontariohealth/files/2020-04/Ontario Health Cancer Care Ontario COVID-19 Supplemental Clinical Guidance for Patients with Cancer_29Mar20 PDF.pdf
131.	ON	Ontario Health	President and CEO Ontario Health	A Measured Approach to Planning for Surgeries and Procedures During the COVID-19 Pandemic	Memo	Publicly available webpage	13-May-20	https://www.ontariohealth.ca/sites/ontariohealth/files/2020-05/A Measured Approach to Planning for Surgeries and Procedures During the COVID-19 Pandemic.pdf

132.	ON	Ontario Ministry of Health	Ontario Ministry of Health	COVID-19 Operational Requirements: Health Sector Restart	Guideline	Publicly available webpage	26-May-20	https://www.cmto.com/wp-content/uploads/2021/11/operational_requirements_health_sector.pdf
133.	ON	Ontario Health	Ontario Health	Infection Prevention and Control (IPAC) for scheduled surgeries and procedures during the COVID-19 pandemic	Guideline	Publicly available webpage	8-Jun-20	https://www.ontariohealth.ca/sites/ontariohealth/files/2020-06/COVID-19 Infection Prevention and Control for Scheduled Surgeries and Procedures_8June2020.pdf
134.	ON	Ontario Health	President and CEO Ontario Health	Updated - A measured Approach to Planning for Surgeries and Procedures During the COVID-19 Pandemic	Memo	Publicly available webpage	15-Jun-20	https://www.ontariohealth.ca/sites/ontariohealth/files/2020-05/A Measured Approach to Planning for Surgeries and Procedures During the COVID-19 Pandemic.pdf
135.	ON	Ontario Health	Ontario Health	Optimizing elective surgery: virtual care supports in the COVID-19 context	Informational /Handout	Publicly available webpage	18-Jun-20	https://www.ontariohealth.ca/sites/ontariohealth/files/2020-06/Surgical Placemat ENGLISH FINAL PDF.pdf
136.	ON	Ontario Health	Ontario Health	Optimizing Care Through COVID-19 Transmission Scenarios:Recommendations from Ontario Health	Guideline	Publicly available webpage	1-Oct-20	https://www.ontariohealth.ca/sites/ontariohealth/files/2020-10/Optimizing Care Through COVID-19 Transmission Scenarios_EN.pdf

137.	ON	Ontario Health	President and CEO Ontario Health	Actions for Optimizing Care Through COVID-19 Transmission Scenarios	Memo	Publicly available webpage	15-Dec-20	https://www.corhealthontario.ca/OH-Memo-Actions-for-Optimizing-Care-(Dec-15).pdf
138.	ON	Ontario Ministry of Health	Acting Chief Medical Officer of Health	COVID-19 Directive #2 for Health Care Providers (Regulated Health Professionals or Persons who operate a Group Practices of Regulated Health Professionals) Issued under Section 77.7 of the Health Protection and Promotion Act (HPPA), r.s.o. 1990, c. H.7	Memo	Publicly available webpage	20-Apr-21	COVID-19 Directive #2 for Health Care Providers (Regulated Health Professionals or Persons who operate a Group Practices of Regulated Health Professionals) Issued under Section 77.7 of the Health Protection and Promotion Act (HPPA), r.s.o. 1990, c. H.7
139.	ON	Ontario Government	Ontario Government	Ontario ramping up efforts to reduce surgical wait times	News Release	Publicly available webpage	28-Jul-21	https://news.ontario.ca/en/release/1000613/ontario-ramping-up-efforts-to-reduce-surgical-wait-times
140.	ON	Ontario Ministry of Health	Chief Medical Officer of Health	Gradual Resumption of Selected Clinical Activities	Memo	Publicly available webpage	1-Feb-22	https://www.health.gov.on.ca/en/pro/programs/publichealth/coronavirus/docs/memos/CMOH_memo_02012022.pdf
141.	ON	Ontario Ministry of Health	Chief Medical Officer of Health	Resuming Non-Urgent and Non-Emergent Care	Memo	Publicly available webpage	10-Feb-22	https://www.health.gov.on.ca/en/pro/programs/publichealth/coronavirus/docs/memos/CMOH_memo_02112022.pdf

142.	ON	Ontario Ministry of Health	Chief Medical Officer of Health	Revocation of CMOH Directives #1, #2.1, #3, #4, and #5	Memo	Publicly available webpage	10-Jun-22	https://www.health.gov.on.ca/en/pro/programs/publichealth/coronavirus/docs/memos/CMOH_memo_06102022.pdf
143.	ON	Guelph General Hospital	Guelph General Hospital	Information for patients undergoing a surgery or procedure during COVID-19	Informational /Handout	Publicly available webpage	1-Aug-22	https://www.gghorg.ca/uncategorized/surgery/information-for-patients-undergoing-a-surgery-or-procedure-during-covid-19/
144.	ON	Ontario Government	Ontario Government	Plan to Stay Open: Health System Stability and Recovery	Plan	Publicly available webpage	18-Aug-22	https://www.ontario.ca/page/plan-stay-open-health-system-stability-and-recovery
145.	ON	Ontario Government	Ontario Government	Fall Preparedness Plan for Health, Long-term care and Education - Keeping Ontarians Safe: Preparing for future waves of COVID-19	Plan	Publicly available webpage	30-Sep-20	https://files.ontario.ca/moh-preparing-for-future-waves-of-covid-19-en-2020-09-30-v1.pdf
146.	ON	Sunnybrook Health Sciences Centre	Sunnybrook Health Sciences Centre	Information for patients undergoing surgery or procedures during COVID-19	Informational /Handout	Publicly available webpage	Undated	https://sunnybrook.ca/content/?page=novel-coronavirus-covid-19-surgery-procedure
147.	ON	Oak Valley Health	Oak Valley Health	Your surgical care journey	Informational /Handout	Publicly available webpage	Undated	https://www.oakvalleyhealth.ca/clinics-departments/surgery/your-surgical-care-journey/
148.	PE	Queen Elizabeth Hospital	Provincial Chief of Surgery	QEH OR Meeting Highlights	Memo	Relevant department	22-Jul-20	NA

149.	PE	Government of Prince Edward Island	Government of Prince Edward Island	Health PEI reducing some surgeries and services to maintain hospital capacity	Report	Publicly available webpage	28-Jan-22	https://www.princeedwardisland.ca/en/news/health-pei-reducing-some-surgeries-and-services-to-maintain-hospital-capacity
150.	PE	Government of Prince Edward Island	Government of Prince Edward Island	Cataract surgeries reinstated, fewer surgical delays expected this week	Report	Publicly available webpage	7-Feb-22	https://www.princeedwardisland.ca/en/news/cataract-surgeries-reinstated-fewer-surgical-delays-expected-this-week
151.	PE	Queen Elizabeth Hospital	Provincial Chief of Surgery	QEH OR AND COVID-19 PLAN	Guideline	Relevant department	27-Mar-20	NA
152.	PE	Queen Elizabeth Hospital	Provincial Chief of Surgery	COVID-19 crisis preparation in the QEH OR	Guideline	Relevant department	31-Mar-20	NA
153.	PE	Queen Elizabeth Hospital	Provincial Chief of Surgery	COVID-19 and the QEH OR	Memo	Relevant department	Undated (March 2020)	NA
154.	PE	Queen Elizabeth Hospital	Provincial Chief of Surgery	Untitled_table showing examples of triage bands	Guideline	Relevant department	Undated (March 2020)	NA
155.	PE	Queen Elizabeth Hospital	Provincial Chief of Surgery	Resumption of high-priority and elective surgeries in PEI during the COVID-19 pandemic	Plan	Relevant department	29-Apr-20	NA
156.	PE	PEI Health	Provincial Chief of Surgery	Surgical Oncology on PEI	Report	Relevant department	10-Jul-20	NA

157.	PE	NSHA	Nova Scotia Health Authority	Decision and management protocol for surgical procedures requiring general anesthesia during COVID-19	Guideline	Relevant department	23-May-22	NA
158.	QC	Ministry of Health and Social Services	Ministry of Health and Social Services	Operating Room Committee-COVID-19	Memo	Relevant department	17-Mar-20	NA
159.	QC	Ministry of Health and Social Services	Information Technology Branch	COVID-19 Bulletin - Telehealth #2	Memo	Relevant department	17-Mar-20	NA
160.	QC	Ministry of Health and Social Services	Ministry of Health and Social Services	Memo_20-MS-00496-30_LET_Bloc opératoire	Memo	Relevant department	20-Mar-20	NA
161.	QC	Ministry of Health and Social Services	Ministry of Health and Social Services	Memo_20-MS-00496-30_PDG_Bloc opératoire	Memo	Relevant department	23-Mar-20	NA
162.	QC	Ministry of Health and Social Services	Ministry of Health and Social Services	Memo_20-MS-00496-83_PDG_Bloc opératoire	Memo	Relevant department	25-Mar-20	NA
163.	QC	Ministry of Health and Social Services	Quebec Cancer Program	Release Plan and Levels of Activities in Cancerology in the Situation of the COVID-19 Pandemic	Guideline	Relevant department	1-Apr-20	NA
164.	QC	Ministry of Health and Social Services	Ministry of Health and Social Services	Memo_20-MS-02502-04_LET_PDG-DSP_Bloc opératoire_Direct_COVID-19	Memo	Relevant department	1-Apr-20	NA

165.	QC	Ministry of Health and Social Services	Ministry of Health and Social Services	Memo_20-MS-02502-33_LET_PDG_Protection of patients and nursing staff	Memo	Relevant department	3-Apr-20	NA
166.	QC	Ministry of Health and Social Services	Ministry of Health and Social Services	Memo_20-MS-02502-52_LET_PDG_Bloc opératoire	Memo	Relevant department	7-Apr-20	NA
167.	QC	Ministry of Health and Social Services	Ministry of Health and Social Services	Recommendations for patient prioritization by tumor site in the context of the COVID-19 pandemic	Guideline	Relevant department	14-Apr-20	NA
168.	QC	Ministry of Health and Social Services	General Directorate of Academic, Medical, Nursing and Pharmaceutical Affairs	Additional Load Shedding 5th Wave	Memo	Relevant department	22-Apr-20	NA
169.	QC	Ministry of Health and Social Services	Ministry of Health and Social Services	Memo_20-MS-02908-96_LET_PDG_Activités spécialisées délestées	Memo	Relevant department	13-May-20	NA
170.	QC	McGill University Health Centre	McGill University Health Centre	COVID-19 Resumption of Activities	Guideline	Relevant department	28-May-20	NA
171.	QC	Ministry of Health and Social Services	COVID-19 Clinical Steering Committee Working Group	Prioritizing Surgery During the COVID-19 Pandemic: the Quebec guidelines	Guideline	Relevant department	12-Jan-21	NA
172.	QC	Ministry of Health and Social Services	Assistant Deputy Minister	5th Wave Surgical theatre load shedding	Memo	Relevant department	20-Dec-21	NA

173.	QC	National Institute of Public Health of Quebec	Committee on Nosocomial Infections of Quebec	Opinion of the Committee on Nosocomial Infections of Quebec: Operation with suspected or confirmed cases of COVID	Guideline	Relevant department	Undated	NA
174.	QC	Universite de Sherbrooke	Universite de Sherbrooke	Management of COVID-19 patient in the operating room	Guideline	Relevant department	Undated	NA
175.	QC	Ministry of Health and Social Services	Ministry of Health and Social Services	Recommendations for intubation in the operating room of confirmed or suspected COVID-19 patients	Guideline	Relevant department	Undated	NA
176.	SK	Saskatchewan Health Authority	Saskatchewan Health Authority	Pandemic Operation Planning Matrix	Guideline	Relevant department	13-Mar-20	NA
177.	SK	Saskatchewan Health Authority	Provincial heads	Cancellation/postponement of non-urgent procedures, clinics, surgeries, appointments, adult day programs in Special Care Homes	Memo	Relevant department	18-Mar-20	NA
178.	SK	Saskatchewan Health Authority	Saskatchewan Health Authority	Unnamed_Flow Chart for Surgery for COVID-19 positive patients	Guideline	Relevant department	19-Mar-20	NA
179.	SK	Saskatchewan Health Authority	Saskatchewan Health Authority	Pre-operative guidelines for suspected or confirmed COVID-19 patients coming to the operating room	Guideline	Relevant department	19-Mar-20	NA

180.	SK	Saskatchewan Health Authority	Saskatchewan Health Authority	Infection Prevention and Control (IPC) Protocol for Surgical Patients During the COVID-19 Pandemic: Adults and Older Adults and Obstetrics	Guideline	Relevant department	9-Apr-20	NA
181.	SK	Saskatchewan Health Authority	Saskatchewan Health Authority	Algorithm for Operative Management of Adult Surgical Patients during COVID-19 Pandemic	Guideline	Relevant department	17-Apr-20	NA
182.	SK	Saskatchewan Health Authority	Director of infection Prevention and Control	Temporary Negative Pressure and Anterooms	Memo	Relevant department	10-Jun-20	NA
183.	SK	Saskatchewan Health Authority	Provincial Infection Prevention and Control	Dismantling of temporary negative pressure rooms due to lack of evidence supporting neccesity	Memo	Relevant department	15-Jun-20	NA
184.	SK	Saskatchewan Health Authority	Saskatchewan Health Authority	Service Reduction Decision Making and Communication	Guideline	Relevant department	19-Oct-20	NA
185.	SK	Saskatchewan Health Authority	Saskatchewan Health Authority	Protocol for Operative Management of Surgical Patients (ALL Ages)	Guideline	Relevant department	30-Nov-20	NA
186.	SK	Saskatchewan Health Authority	Lori Garchinski, Executive Director, Provincial Programs, and Petrina McGrath and Dr. Michael Kelly, EOC Safety Officers	Airway/protected CODE BLUE response plan for patients during COVID-19 pandemic	Memo	Relevant department	1-Dec-20	NA

187.	SK	Saskatchewan Health Authority	Saskatchewan Health Authority	Protocol for Operative Management of Surgical Patients (ALL Ages)_updated	Guideline	Relevant department	22-Dec-20	NA
188.	SK	Saskatchewan Health Authority	Saskatchewan Health Authority	Surgical Response to the New Vaccine Framework	Memo	Relevant department	16-Feb-21	NA
189.	SK	Saskatchewan Health Authority	Saskatchewan Health Authority	Recommendations for delaying elective surgery for patients recovering from COVID-19	Guideline	Relevant department	3-Jun-21	NA
190.	SK	University of Saskatchewan and Saskatchewan Health Authority	University of Saskatchewan and Saskatchewan Health Authority	Department of Surgery 4th Wave COVID-19 Guidelines: 3-week & 6-week Urgent Cases	Guideline	Relevant department	6-Oct-21	NA
191.	SK	Government of Saskatchewan	Government of Saskatchewan	Province announces plans to eliminate COVID-19 surgical backlog and expand ICU	News Release	Publicly available webpage	9-Dec-21	https://www.saskatchewan.ca/government/news-and-media/2021/december/09/province-announces-plans-to-eliminate-covid-surgical-backlog-and-expand-icu
192.	SK	Government of Saskatchewan	Government of Saskatchewan	Surgical Performance and Wait Times	News Release	Publicly available webpage	26-May-22	https://www.saskatchewan.ca/residents/health/accessing-health-care-services/surgery/surgical-performance-and-wait-times
193.	SK	Saskatchewan Health Authority	Saskatchewan Health Authority	Frequently Asked Questions: COVID-19 Testing Before Surgery	Informational /Handout	Relevant department	1-Jul-22	NA

194.	SK	Government of Saskatchewan	Government of Saskatchewan	Surgical Update - Backgrounder	News Release	Publicly available webpage	1-Jul-22	https://www.saskatchewan.ca/-/media/news-release-backgrounders/2022/jul/surgical-status-update.pdf
195.	SK	Saskatchewan Health Authority	Saskatchewan Health Authority	COVID-19 Health Service Monitoring and Reduction Framework	Guideline	Relevant department	6-Nov-20	NA
196.	SK	Ministry of Health	Ministry of Health	Ministry of Health Business Plan 2022-23	Plan	Publicly available webpage	Undated 2022	https://pubsaskdev.blob.core.windows.net/pubsask-prod/134520/HealthPlan2022-23.pdf
197.	SK	Saskatchewan Health Authority	Saskatchewan Health Authority	Annual Report to the Legislature 2021 - 2022	Report	Publicly available webpage	Undated 2022	https://www.saskhealthauthority.ca/sites/default/files/2022-07/Report-CEC-SHA-Annual-2021-22.pdf
198.	SK	Saskatchewan Health Authority	Saskatchewan Health Authority	Putting Patients First: Eliminating the Surgical Backlog	Plan	Publicly available webpage	Undated	https://www.saskatchewan.ca/-/media/news-archive/2009/october/29/work-begins-on-improving-surgical-care-and-reducing-wait-times/surgical-care-and-wait-times.pdf
199.	SK	Government of Saskatchewan	Government of Saskatchewan	Targets to Address Surgical Backlog and ICU expansion	Plan	Publicly available webpage	31-Dec-21	https://www.saskatchewan.ca/-/media/news-release-backgrounders/2021/dec/service-resumption-backgrounder.pdf

200.	SK	University of Saskatchewan and Saskatchewan Health Authority	University of Saskatchewan and Saskatchewan Health Authority	COVID-19 Evidence Support Team Rapid Review Report: What is the evidence on timing and outcomes of elective surgery after a COVID infection?	Report	Relevant department	Undated	NA
201.	SK	Saskatchewan Health Authority	Saskatchewan Health Authority	COVID-19 Testing Prior to Surgery	Informational /Handout	Relevant department	Undated	NA
202.	YT	Yukon Hospital Corporation	Yukon Hospital Corporation	COVID-19 Updates	Informational /Handout	Publicly available webpage	24-Oct-22	https://yukonhospitals.ca/en/about-us/covid-19-updates
203.	Federal	Government of Canada	Department of Finance	Canada commits $2 billion in additional health care funding to clear backlogs and support hundreds of thousands of additional surgeries	News Release	Publicly available webpage	25-Mar.2022	https://www.canada.ca/en/department-finance/news/2022/03/canada-commits-2-billion-in-additional-health-care-funding-to-clear-surgery-and-diagnostics-backlogs.html
204.	Federal	Canadian Institute for Health Information	Canadian Institute for Health Information	Canadian COVID-19 intervention timeline	Report	Publicly available webpage	13-Oct-22	https://www.cihi.ca/en/canadian-covid-19-intervention-timeline
205.	Federal	Government of Canada	Government of Canada	COVID-19 epidemiology update	Report	Publicly available webpage	30-Oct-22	COVID-19 epidemiology update: Key updates — Canada.ca Canada.ca · https://health-infobase.canada.ca › covid-19
