# Supplementary material for: Delivery and Prioritization of Surgical Care in Canada During COVID-19: An Environmental Scan
Source: Int J Health Policy Manag. 2023 Dec 10;12:8007. doi: 10.34172/ijhpm.2023.8007 (PMC10843432; doi:10.34172/ijhpm.2023.8007)
Supplement: Supplementary file 3 — contains Table S2. [file ijhpm-12-8007-s003.pdf]

Article title: Delivery and Prioritization of Surgical Care in Canada During COVID-19: An Environmental Scan

Journal name: International Journal of Health Policy and Management (IJHPM)

Authors' information: Seremi Ibadin[1], Mary Brindle[2,3], Tracy Wasylak[3], Jill Robert[4], Stacey Litvinchuk[3], Khara M. Sauro[1,2,5]*

[1]Department of Community Health Sciences, Cumming School of Medicine, University of Calgary, Calgary, AB, Canada.

[2]Department of Surgery, Cumming School of Medicine, University of Calgary, Calgary, AB, Canada.

[3]Surgery Strategic Clinical Networks, Alberta Health Services, Calgary, AB, Canada.

[4]Surgery and Bone & Joint Health Strategic Clinical Networks, Alberta Health Services, Calgary, AB, Canada.

[5]Department of Oncology and Arnie Charbonneau Cancer Institute, Cumming School of Medicine, University of Calgary, Calgary, AB, Canada.

***Correspondence to:** Khara M. Sauro; Email: kmsauro@ucalgary.ca

Citation: Ibadin S, Brindle M, Wasylak T, Robert J, Litvinchuk S, Sauro KM. Delivery and prioritization
of surgical care in Canada during COVID-19: an environmental scan. Int J Health Policy Manag. 2023;12:8007. doi:10.34172/ijhpm.2023.8007

Supplementary file 3

Table S2: Guidance for Prioritization of Surgical Cases During COVID-19 across Canada

**Province**	**Guidance for surgical activity/prioritization**	**Summary**	**Guidance for cancer surgery prioritization**
Alberta	Surgical Services: Pandemic Planning & Mitigation Strategies - Framework for Surgical Services Slowdowns (December 2020)	Level 1 - 100%, all procedures proceed including emergent, urgent, and scheduled categories<br>Level 2 - 30 % reduction of elective surgery if required<br>Level 3 - 60% reduction in all activity, continued emergent and	COVID-19 Planning Clinical Guidance for Patients with Cancer (March 2020)*<br><br>Cancer Surgery Pandemic Protocol (updated September 2021)

	Alberta Pandemic Surgery Resumption Framework: A Framework to Support Graduated Approach to Surgical Service Reinstatement (0ctober 2021)<br><br>Alberta Coding Access Targets for Surgery (ACATS)	urgent (< 7 days), cancer triaging guidelines applied<br>Level 4 **-** Up to 75% reduction in overall activity, cancer triaging guidelines applied, limb procedures only, emergent only (< 3 days)<br>Maintain activity at chartered surgical facilities (Level 1 – 4) unless directed by Chief Medical Officer of Health<br>ACATS codes used to support patient prioritization	
British Columbia	Criteria for Clinical Prioritization During the COVID-19 Pandemic (March 2020)*		Provincial Cancer Clinical Management guidelines in Pandemic (April 2020)<br>BC Cancer Tumor Group Specific Prioritization and Mitigation Recommendations during COVID-19 Pandemic (May 2020)
Manitoba	Restart MB Pandemic Response System (January 2021)	Recommendations for surgical activity/prioritization based on pandemic response levels (Green, Yellow, Orange Red)<br>Green (Limited risk) - Very low virus transmission levels, sufficient health system capacity - no extraordinary public health measures required<br>Yellow (Caution) - Spread of COVID-19 is at low to moderate	Clinical Guidelines for Prioritizing Cancer Services in Manitoba during COVID-19 Pandemic.*

		levels - public health measures, but no restriction on health services<br>Orange (Restricted) - Community transmission of COVID-19 is occurring across much of Manitoba, health care system can manage COVID-19 case levels - non-urgent and elective diagnostic services, surgeries and procedures may be limited according to human resource capacity, physical distancing, and personal protective equipment requirements. If clinical activity is affected, care will be prioritized by urgency.<br>Red (Critical) - Extensive community transmission of COVID-19 is occurring and is not contained - non-urgent and elective diagnostic services, surgeries and procedures are limited according to human resource capacity, physical distancing, and personal protective equipment requirements. Clinical activity will be prioritized by urgency. Case-by-case clinical assessment at facility level to prioritize urgent cases for surgery.	

Nova Scotia	NSHA Perioperative and Interventional Radiology Services During COVID-19 Pandemic: Recommendations for Triage of Urgent, Benign and Cancer Surgery (March 2020)	A four-level, priority band approach for triage of procedures based on the American College Surgeons Guidelines for non-emergent cases during COVID-19 pandemic.<br><br>Priority Bands<br>Band 1 - Conditions with threat to life/limb/organ over next 24 hours.<br><br>Band 2 - Conditions with threat to life/organ within two weeks.<br><br>Band 3 - Conditions with threat to progress to emergency within four to eight weeks (for non-cancer cases); conditions with threat to life over next 4 weeks (for cancer cases)<br><br>Band 4 - Conditions where delay of 8 weeks is unlikely to adversely impact outcome (for non-cancer cases); conditions where delay of 8 weeks unlikely to impact oncologic outcome (for cancer cases). These cases are not to be performed during the COVID-19 outbreak	NSHA Perioperative and Interventional Radiology Services During COVID-19 Pandemic: Recommendations for Triage of Urgent, Benign and Cancer Surgery (March 2020)
Ontario	Clinical Triage Protocol for Major Surge in COVID Pandemic (March 2020)	Transmission Scenarios:<br>Scenario 1 (No cases) - Resume/accelerate scheduled	Pandemic Planning Clinical Guideline for Patients with Cancer

	A Measured Approach to Planning for Surgeries and Procedures during the COVID-19 Pandemic (May 2020, updated June 2020)<br>Optimizing Care Through COVID-19 Transmission Scenarios: Recommendations from Ontario Health (October 2020)<br>Wait Times Information System (WTIS)	care, strengthen surge plans (all sectors), review and reprioritize waitlists, address time-sensitive care backlog, plan for COVID-protected wards where feasible (hospital-based care)<br>Scenario 2 (sporadic cases) - Resume/accelerate scheduled care, ready surge plans (all sectors), review and reprioritize waitlists, address time-sensitive care backlog (hospital-based care)<br>Scenario 3 (clusters of cases) - maintain/accelerate scheduled care if there is adequate capacity, implement and enhance surge plans (all sectors), create capacity (hospital-based care)<br>Scenario 4A and 4B (moderate community transmission and widespread community transmission) - prioritize time-sensitive surgeries and procedures, consider deferring non-time sensitive surgeries and procedures, implement COVID-protected wards, where feasible<br>Surgical Patients' Population Priorities A, B, C<br>Priority A – delay will result in immediate threat to life or limb, or significantly alter prognosis,	(March 2020, updated December 2021)<br>COVID-19 Supplemental Clinical Guidance for Patients with Cancer (March 2020, Updated December 2021)

		Priority B – delay of < 4 weeks will not be anticipated to have significant impact on survival or outcome,<br>Priority C – delay of 2 months will be unlikely to affect outcome<br><br>WTIS used to support patient prioritization	
Prince Edward Island	Pandemic: Recommendations for Triage of Urgent, Benign and Cancer Surgery (March 2020) adopted from Nova Scotia	A four-level, priority band approach for triage of procedures based on the American College Surgeons Guidelines for non-emergent cases during COVID-19 pandemic.<br><br>Priority Bands<br>Band 1 - Conditions with threat to life/limb/organ over next 24 hours.<br><br>Band 2 - Conditions with threat to life/organ within two weeks.<br><br>Band 3 - Conditions with threat to progress to emergency within four to eight weeks (for non-cancer cases); conditions with threat to life over next 4 weeks (for cancer cases)<br><br>Band 4 - Conditions where delay of 8 weeks is unlikely to	

		adversely impact outcome (for non-cancer cases); conditions where delay of 8 weeks unlikely to impact oncologic outcome (for cancer cases). These cases are not to be performed during the COVID-19 outbreak	
Quebec	Prioritizing Surgery During the COVID-19 Pandemic: the Quebec Guidelines	Overall prioritization of patients awaiting surgery based on pandemic response and recommended activity level<br>Level 1 (Maintain 70% to 100% of OR capacity) – When operating room resources increase, add 10% of less urgent patients per week based on their wait time for surgery<br>Level 2 (Maintain 50% to 70% of OR capacity) - Prioritize patients according to: Those whose survival is most at risk; those nearest to or closest to the maximum surgery date; those requiring the least amount of operating time and resources possible; those with good chance to recover quickly (low to medium American Society of Anesthesiologists (ASA) classification)<br>Level 3 (Maintain 30% to 50% of OR capacity) - Prioritize patient according to: Those whose survival is most at risk;	Load shedding plans and levels of activity in cancerology in the situation of the COVID-19 pandemic (April 2020)<br>Recommendations by tumor site for patient prioritization in the context of COVID-19 (April 2020)

		those nearest to or beyond the maximum surgery date; those requiring the least amount of operating time and resources possible (considering the cases most at risk and requiring more operating time.); those most likely to recover quickly (low ASA classification)<br>Level 4 (Maintain <30% OR capacity) – Emergency cases only	
Saskatchewan	Service Reduction Decision Making and Communication (October 2020)<br><br>Department of Surgery 4th Wave COVID-19 Guidelines: 3-week and 6-week Urgent Cases (October 2021)	Services prioritized according to necessity and patient risk; service reductions will be targeted and not across the board. Urgent and emergent care to continue throughout the pandemic, including services not considered 'elective' such as cancer diagnosis and care<br>Algorithm for decision making based on key principles for service reduction, and triggers for consideration (number of active cases and test positivity rate within network/small geographic area over a period of 7 days, patient facility occupancy, workforce measures, PPE, space, and supply availability.	

*Document title found through internet searches, but authors were unable to retrieve the document for analysis
