# Supplementary material for: Delivery and Prioritization of Surgical Care in Canada During COVID-19: An Environmental Scan
Source: Int J Health Policy Manag. 2023 Dec 10;12:8007. doi: 10.34172/ijhpm.2023.8007 (PMC10843432; doi:10.34172/ijhpm.2023.8007)
Supplement: Supplementary file 4 — contains Table S3. [file ijhpm-12-8007-s004.pdf]

Article title: Delivery and Prioritization of Surgical Care in Canada During COVID-19: An Environmental Scan

Journal name: International Journal of Health Policy and Management (IJHPM)

Authors' information: Seremi Ibadin[1], Mary Brindle[2,3], Tracy Wasylak[3], Jill Robert[4], Stacey Litvinchuk[3], Khara M. Sauro[1,2,5]*

[1]Department of Community Health Sciences, Cumming School of Medicine, University of Calgary, Calgary, AB, Canada.

[2]Department of Surgery, Cumming School of Medicine, University of Calgary, Calgary, AB, Canada.

[3]Surgery Strategic Clinical Networks, Alberta Health Services, Calgary, AB, Canada.

[4]Surgery and Bone & Joint Health Strategic Clinical Networks, Alberta Health Services, Calgary, AB, Canada.

[5]Department of Oncology and Arnie Charbonneau Cancer Institute, Cumming School of Medicine, University of Calgary, Calgary, AB, Canada.

***Correspondence to:** Khara M. Sauro; Email: kmsauro@ucalgary.ca

Citation: Ibadin S, Brindle M, Wasylak T, Robert J, Litvinchuk S, Sauro KM. Delivery and prioritization of surgical care in Canada during COVID-19: an environmental scan. Int J Health Policy Manag. 2023;12:8007. doi:10.34172/ijhpm.2023.8007

Supplementary file 4

Table S3: List of Evidence Sources Containing IPAC and Safety Protocols, Guidance, Recommendations, and Information

S/N	Province/ Territory	Institution	Document Author	Document Title	Source	Document Date	Web Link
1.	AB	Alberta Health Services	Alberta Health Services	IPC Recommendations for suspected or confirmed COVID-19 Patients Requiring Surgery	Publicly available webpage	18-Apr-20	https://www.albertahealthservices.ca/assets/healthinfo/ipc/hi-ipc-suspect-conf-emergency-urgent-surgery.pdf
2.	AB	Alberta Health Services	Alberta Health Services	Point-of-care risk assessment for Surgery during COVID-19	Publicly available webpage	8-Dec-20	https://www.albertahealthservices.ca/assets/healthinfo/ipc/hi-ipc-covid-19-or-algorithm.pdf
3.	AB	Alberta Health Services	Alberta Health Services	IPC PPE table for surgical suites	Publicly available webpage	17-Dec-20	https://www.albertahealthservices.ca/assets/healthinfo/ipc/hi-ipc-ppe-tbl-surg-covid-19.pdf
4.	AB	Alberta Health Services	Alberta Health Services (Surgery Strategic Clinical Network)	Guidance for surgery after COVID-19 infection: Timing of surgery following recovery from COVID-19 TO reduce risk of postoperative mortality	Publicly available webpage	20-Jan-22	https://www.albertahealthservices.ca/assets/info/ppih/if-ppih-covid-19-guidance-surgery-after-covid-19.pdf

5.	BC	BC Ministry of Health	BC Ministry of Health	Options for Operating Room Configuration and Use When a Patient with Suspected or Confirmed COVID-19 Requires Emergent Surgery	Publicly available webpage	12-Jan-22	http://www.bccdc.ca/Health-Professionals-Site/Documents/COVID19_ORConfigUseEmergentSurgery.pdf
6.	BC	BC Ministry of Health	BC Ministry of Health	Infection Prevention and Control (IPC) Protocol for Adult Surgical Procedures During the Covid-19 Pandemic	Publicly available webpage	11-May-22	http://www.bccdc.ca/Health-Professionals-Site/Documents/COVID19_IPCProtocolSurgicalProceduresAdult.pdf
7.	BC	BC Ministry of Health	BC Ministry of Health	Infection Prevention and Control (IPC) Protocol for Paediatric Surgical Procedures During the Covid-19 Pandemic	Publicly available webpage	Undated	http://www.bccdc.ca/Health-Professionals-Site/Documents/COVID19_IPCProtocolSurgicalProceduresPediatric.pdf
8.	BC	BC Ministry of Health	BC Ministry of Health	COVID-19 and Adult Surgeries: How We Are Keeping You Safe	Publicly available webpage	Undated	http://www.bccdc.ca/Health-Professionals-Site/Documents/COVID-19_Adult_Surgical_Patient_Handout.pdf

9.	BC	BC Ministry of Health	BC Ministry of Health	COVID-19 and Paediatric Procedure: How We Are Keeping Your Child Safe	Publicly available webpage	Undated	http://www.bccdc.ca/Health-Professionals-Site/Documents/COVID-19_Pediatric_Surgical_Patient_Handout.pdf
10.	MB	Shared Health	Shared Health	COVID-19 Operating Room Risk Stratification for Surgical Patients	Publicly available webpage	23-Feb-21	https://sharedhealthmb.ca/files/covid-19-risk-stratification-for-surgical-patients.pdf
11.	MB	Shared Health	Shared Health	Provincial Guidance for COVID-19: A Return to Symptomatic Testing Infection Prevention and Control Risk and Response	Publicly available webpage	15-Jun-22	https://sharedhealthmb.ca/files/covid-19-admission-preop-testing.pdf
12.	NB	Robert Wood Johnson University Hospital	Robert Wood Johnson University Hospital	Safe surgery during COVID-19 at RWJUH New Brunswick: Keeping everyone safe	Publicly available webpage	Undated	https://www.rwjbh.org/rwj-university-hospital-new-brunswick/patients-visitors/temporary-changes-to-services-and-visitation-pol/safe-surgery-during-covid-19/

13.	NB	Vitale Health	Vitale Health	Preparing for surgery (Orange level)	Publicly available webpage	Undated	https://www.vitalitenb.ca/en/covid-19/preparing-for-surgery
14.	NL	Eastern Health	Eastern Health	Strategic Health Plan 2020 - 2023	Publicly available webpage	Undated	https://www.easternhealth.ca/wp-content/uploads/2021/05/Eastern-Health_Strategic-Plan_2020-23.pdf
15.	NS	Nova Scotia Health Authority	Nova Scotia Health Authority	Surgical services at Aberdeen Hospital to reopen April 11	Publicly available webpage	9-Apr-20	https://nshealth.ca/news/surgical-services-aberdeen-hospital-reopen-april-11
16.	NS	Nova Scotia Health Authority	Nova Scotia Health Authority	NSHA addresses "community clusters" document on social media	Publicly available webpage	29-Apr-20	https://www.nshealth.ca/news/nsha-addresses-community-clusters-document-social-media - :~:text=NSHA addresses "community clusters" document on social media,-Wednesday, April 29&text=We are aware of Nova,or spread of COVID-19.

17.	NS	Nova Scotia Health Authority	Krista Woods	COVID-19 requires teams to rethink how they deliver surgical care	Publicly available webpage	18-Dec-20	https://www.nshealth.ca/news/covid-19-requires-teams-rethink-how-they-deliver-surgical-care
18.	NS	Nova Scotia Health Authority	Nova Scotia Health Authority	Decision and Management Protocol for Surgical Procedures Requiring Anesthesia during COVID-19	Publicly available webpage	23-May-22	https://policy.nshealth.ca/Site_Published/covid19/document_render.aspx?documentRender.IdType=6&documentRender.GenericField=&documentRender.Id=83387
19.	NT	Northwest Territories Health and Social Services Authority	Northwest Territories Health and Social Services Authority	COVID Testing	Publicly available webpage	Undated	https://www.nthssa.ca/en/covid-testing - :~:text=Tests are available in your community%3A&text=COVID-19 testing to inform,ordered by a healthcare provider.
20.	ON	Ontario Health	Ontario Health	Infection Prevention and Control (IPAC) for scheduled surgeries and procedures during the COVID-19 pandemic	Publicly available webpage	8-Jun-20	https://www.ontariohealth.ca/sites/ontariohealth/files/2020-06/COVID-19 Infection Prevention and Control for Scheduled

							Surgeries and Procedures_8June2020.pdf
21.	ON	Guelph General Hospital	Guelph General Hospital	Information for patients undergoing a surgery or procedure during COVID-19	Publicly available webpage	1-Aug-22	https://www.gghorg.ca/uncategorized/surgery/information-for-patients-undergoing-a-surgery-or-procedure-during-covid-19/
22.	ON	Sunnybrook Health Sciences Centre	Sunnybrook Health Sciences Centre	Information for patients undergoing surgery or procedures during COVID-19	Publicly available webpage	Undated	https://sunnybrook.ca/content/?page=novel-coronavirus-covid-19-surgery-procedure
23.	ON	Oak Valley Health	Oak Valley Health	Your surgical care journey	Publicly available webpage	Undated	https://www.oakvalleyhealth.ca/clinics-departments/surgery/your-surgical-care-journey/
24.	PE	Queen Elizabeth Hospital	Provincial Chief of Surgery	QEH OR Meeting Highlights	Relevant department	22-Jul-20	NA

25.	PE	Queen Elizabeth Hospital	Provincial Chief of Surgery	COVID-19 crisis preparation in the QEH OR	Relevant department	31-Mar-20	NA
26.	PE	Nova Scotia Health Authority	Nova Scotia Health Authority	Decision and management protocol for surgical procedures requiring general anesthesia during COVID-19	Relevant department	23-May-22	NA
27.	QC	Ministry of Health and Social Services	Ministry of Health and Social Services	Memo_20-MS-00496-83_PDG_Bloc opératoire	Relevant department	25-Mar-20	NA
28.	QC	Ministry of Health and Social Services	Ministry of Health and Social Services	Memo_20-MS-02502-04_LET_PDG-DSP_Bloc opératoire_Direct_COVID-19	Relevant department	1-Apr-20	NA
29.	QC	Ministry of Health and Social Services	Ministry of Health and Social Services	Memo_20-MS-02502-52_LET_PDG_Bloc opératoire	Relevant department	7-Apr-20	NA
30.	QC	National Institute of Public Health of Quebec	Committee on Nosocomial Infections of Quebec	Opinion of the Committee on Nosocomial Infections of Quebec: Operation with suspected or confirmed cases of COVID	Relevant department	Undated	NA

31.	QC	Universite de Sherbrooke	Universite de Sherbrooke	Management of COVID-19 patient in the operating room	Relevant department	Undated	NA
32.	QC	Ministry of Health and Social Services	Ministry of Health and Social Services	Recommendations for intubation in the operating room of confirmed or suspected COVID-19 patients	Relevant department	Undated	NA
33.	SK	Saskatchewan Health Authority	Saskatchewan Health Authority	Unnamed_Flow Chart for Surgery for COVID-19 positive patients	Relevant department	19-Mar-20	NA
34.	SK	Saskatchewan Health Authority	Saskatchewan Health Authority	Pre-operative guidelines for suspected or confirmed COVID-19 patients coming to the operating room	Relevant department	19-Mar-20	NA
35.	SK	Saskatchewan Health Authority	Saskatchewan Health Authority	Infection Prevention and Control (IPC) Protocol for Surgical Patients During the COVID-19 Pandemic: Adults and Older Adults and Obstetrics	Relevant department	9-Apr-20	NA
36.	SK	Saskatchewan Health Authority	Saskatchewan Health Authority	Algorithm for Operative Management of Adult Surgical Patients during COVID-19 Pandemic	Relevant department	17-Apr-20	NA

37.	SK	Saskatchewan Health Authority	Director of infection Prevention and Control	Temporary Negative Pressure and Anterooms	Relevant department	10-Jun-20	NA
38.	SK	Saskatchewan Health Authority	Provincial Infection Prevention and Control	Dismantling of temporary negative pressure rooms due to lack of evidence supporting neccesity	Relevant department	15-Jun-20	NA
39.	SK	Saskatchewan Health Authority	Saskatchewan Health Authority	Protocol for Operative Management of Surgical Patients (ALL Ages)	Relevant department	30-Nov-20	NA
40.	SK	Saskatchewan Health Authority	Lori Garchinski, Executive Director, Provincial Programs, and Petrina McGrath and Dr. Michael Kelly, EOC Safety Officers	Airway/protected CODE BLUE response plan for patients during COVID-19 pandemic	Relevant department	1-Dec-20	NA
41.	SK	Saskatchewan Health Authority	Saskatchewan Health Authority	Protocol for Operative Management of Surgical Patients (ALL Ages)_updated	Relevant department	22-Dec-20	NA

42.	SK	Saskatchewan Health Authority	Saskatchewan Health Authority	Surgical Response to the New Vaccine Framework	Relevant department	16-Feb-21	NA
43.	SK	Saskatchewan Health Authority	Saskatchewan Health Authority	Recommendations for delaying elective surgery for patients recovering from COVID-19	Relevant department	3-Jun-21	NA
44.	SK	Saskatchewan Health Authority	Saskatchewan Health Authority	Frequently Asked Questions: COVID-19 Testing Before Surgery	Relevant department	1-Jul-22	NA
45.	SK	Saskatchewan Health Authority	Saskatchewan Health Authority	COVID-19 Testing Prior to Surgery	Relevant department	Undated	NA
46.	SK	University of Saskatchewan and Saskatchewan Health Authority	University of Saskatchewan and Saskatchewan Health Authority	COVID-19 Evidence Support Team Rapid Review Report: What is the evidence on timing and outcomes of electove surgery after a COVID infection?	Relevant department	Undated	NA
