# Supplementary material for: Delivery and Prioritization of Surgical Care in Canada During COVID-19: An Environmental Scan
Source: Int J Health Policy Manag. 2023 Dec 10;12:8007. doi: 10.34172/ijhpm.2023.8007 (PMC10843432; doi:10.34172/ijhpm.2023.8007)
Supplement: Supplementary file 5 — contains Table S4. [file ijhpm-12-8007-s005.pdf]

Article title: Delivery and Prioritization of Surgical Care in Canada During COVID-19: An Environmental Scan

Journal name: International Journal of Health Policy and Management (IJHPM)

Authors' information: Seremi Ibadin[1], Mary Brindle[2,3], Tracy Wasylak[3], Jill Robert[4], Stacey Litvinchuk[3], Khara M. Sauro[1,2,5]*

[1]Department of Community Health Sciences, Cumming School of Medicine, University of Calgary, Calgary, AB, Canada.

[2]Department of Surgery, Cumming School of Medicine, University of Calgary, Calgary, AB, Canada.

[3]Surgery Strategic Clinical Networks, Alberta Health Services, Calgary, AB, Canada.

[4]Surgery and Bone & Joint Health Strategic Clinical Networks, Alberta Health Services, Calgary, AB, Canada.

[5]Department of Oncology and Arnie Charbonneau Cancer Institute, Cumming School of Medicine, University of Calgary, Calgary, AB, Canada.

***Correspondence to:** Khara M. Sauro; Email: kmsauro@ucalgary.ca

Citation: Ibadin S, Brindle M, Wasylak T, Robert J, Litvinchuk S, Sauro KM. Delivery and prioritization

of surgical care in Canada during COVID-19: an environmental scan. Int J Health Policy Manag. 2023;12:8007. doi:10.34172/ijhpm.2023.8007

Supplementary file 5

Table S4: Provincial Surgery Recovery Plans

Recovery Plans and Initiatives	Details
**Alberta**<br>○ Surgery Recovery Plan (June 2020)<br>○ COVID-19 Pandemic Recovery Playbook (October 2021)<br>○ Alberta Provincial Surgery Recovery Plan: Application of the Alberta Surgical Initiative as the Framework for Pandemic Recovery and Surgery Wait time Reduction (April 2022)	**Goal** - All Albertans receive scheduled surgeries within a clinically appropriate timeframe by 2023.<br>**Strategies**<br>Short to medium-term strategies to reduce backlogs<br>• Increase volume of surgeries at chartered surgical facilities<br>• Establish new, publicly funded contracts to expand the scope of surgeries<br>• Create focused sites of surgical care<br>Strategies outlined within the Alberta Surgical Initiative's 5-pronged approach serve as the framework for surgery recovery and targeted waitlist reduction. Highlights include:<br>• Increase patient engagement and communication<br>• Understand and shape demand for surgery by managing appropriate and timely surgical referrals and waitlist management<br>• Optimize and increase surgical capacity – establish provincial OR utilization benchmark, increase utilization of Chartered Surgical Facilities, increase recruitment and training of the

	highly skilled workforce required for surgery recovery, optimize surgical capacity for complex surgeries, implement patient-centred and equitable surgical load leveling across Zones<br>• Improve surgical care pathways to reduce overall reliance on acute care beds, expand utilization of day surgeries for eligible cases, reduce in-patient duration of stay<br>• Ensure monitoring and accountability<br>**Provincial Recovery Targets (as of April 2022)[a]**<br>Recovery metrics – total number of patients waiting for scheduled surgery, number waiting out of target, % waiting out of target, total surgical volume<br>Targets<br>• Total wait list for scheduled surgery reduced to ~56,000 or less (from pre-pandemic baseline of 68,000 and 2021/22 estimate of 79,090<br>• Reduction of patients waiting out of target to ~21,000 (38%) or less from pre-pandemic estimate of 27,200 (40%) and 2021/22 estimate of 44,598 (56.4%)<br>• Approximate total surgeries of 336,900 (from baseline of 285,000 in 2018/19 fiscal year and 265,500 in 2021/2022)<br>**Funding**<br>• Province announced commitment of $100 million to renovate, equip and open new operating rooms in urban and rural public hospitals in September 2020<br>• Provincial Budget 2021 included $120 million in operating funds for the Alberta Surgical Initiative to increase access to and number of surgical procedures, plus unspecified additional funding from the $1.25 billion COVID Contingency Fund to address surgical backlogs<br>• Provincial Budget 2022 committed $133 million over 3 years for the ASI Capital Program to increase surgical capacity and ensure all patients receive required surgeries within clinically recommended timelines.<br>• Alberta received a budgetary allocation of $232,332,000 in 2022 from the Federal Government towards clearing surgical backlogs<br>**Reported progress* –** As of September 9, 2022, AHS reported average weekly volume for surgical activity was 92 per cent of pre-pandemic surgical volumes. The surgical wait list for adults was approximately 73,473 (baseline 68,000), and approximately 101,500 surgeries had been completed in the 2022/23 fiscal year (started April 2022).
**British Columbia**<br>○ A Commitment to Surgical Renewal in British Columbia	**Goal -** Significantly increase the number of surgeries performed beyond pre-COVID-19 levels to keep up with new demands for surgery and complete the surgeries lost to COVID-19 (estimated at 30,000 as of May 8, 2020) within 17 to 24 months.<br>**Strategies:**<br>Five steps for delivering surgical renewal:

○ Provincial Health Service Authority 2020/2021 – 2022/2023 Service Plan	• Increase surgeries through generating efficiencies, extending hours, operating on weekends, optimizing operations over traditional slowdown periods, and opening new or unused operating rooms, increase capacity at private surgical facilities for publicly funded surgeries<br>• Increase essential personnel through focused recruitment, additional training, and evaluation and implementation of new models of care.<br>• Focus on patients by calling all patients who had their surgery postponed due to COVID-19 or were waitlisted prior to the resumption of surgeries to confirm they are still willing and able to come for surgery, and by prioritizing patients whose surgeries must occur in less than four weeks; patients who have had their surgery postponed; patients who have waited more than twice their targeted wait time; and patients whose surgeries can safely be conducted as day procedures or outside of the main operating room.<br>• Add more resources<br>• Report on the progress of these efforts.<br>**Funding**<br>• In 2021-22, the provincial government allocated up to $187.5 million to support efforts for surgical renewal.<br>• BC received a budgetary allocation of $272,434,000 in 2022 from the Federal Government towards clearing surgical backlogs.<br>**Reported progress***<br>In May 2022, BC Ministry of Health reported that pre-pandemic surgical volumes had been exceeded in the 2nd year of delivering on its surgical renewal commitments.<br>Over 337,000 surgeries were completed in the 12-month period ended March 31, 2022. Specifically, 99.8% of cases postponed during first wave had their surgeries completed. Surgery completion was 96.2% for 2nd and 3rd wave, and 78.9% for 4th and 5th wave postponements. Reported percentages are based on only patients who still opted to receive surgical treatment following cancellation of scheduled surgeries. The provincial surgery waitlist had decreases by 11.4% compared to peak waitlist size in May 2020.
**Manitoba**<br>○ Diagnostic and Surgery Recovery Task Force (established December 2021)<br>○ Manitoba Virtual Care Action Plan (June 2022)	**Strategies**<br>• Increase surgical capacity through:<br>i. Request for Supply Agreements (RFSA) - contracts were awarded to public and private surgical facilities to address the backlog for urologic procedures, orthopaedic and foot and hand surgeries in August 2020.<br>ii. Create additional capacity (OR, hospital beds and staff) and expand surgery program at selected hospitals<br>iii. Out-of-province partnerships with private surgical facilities to allow Manitobans undergo selected orthopaedic procedures at three facilities (in Ontario, Ohio, and North Dakota) as an interim measure starting in 2022

	• Create a Provincial Surgery Waitlist and Information Management System through the Manitoba Virtual Care Action Plan<br><br>The Manitoba Government established a Diagnostic and Surgical Recovery Task Force in December 2021 to address waitlists for diagnostic and surgical procedures, as well as related services affected by the COVID-19 pandemic.<br>**Funding**<br>• Manitoba Government invested $2.5 million in RFSA as of August 2020, funding commitments for agreements with public and private service providers was estimated at $8.8 million by January 2021.<br>• The Provincial Budget 2021 committed a minimum of $50 million in new funding to reduce wait times for priority surgeries delayed by the pandemic, as well as hip and knee procedures for an expected increase in capacity by up to 1,000 surgeries per year<br>• In Budget 2022, the Manitoba Government announced $110 million to address surgical and diagnostic waitlists through the Task Force.<br>• In June 2022, Health Science Centre (HSC) Foundation launched “Operation Excellence”, a $100 million 6-year capital plan to develop surgical and diagnostic capacity at HSC by 25% over pre-pandemic levels. The provincial government announced an additional commitment of $50 million and the HSC launched a campaign to raise $50 million.<br>• $2.3 million to develop the Provincial Surgery Waitlist and Information Management System (including federal support of 389.4k) announced in June 2022.<br>• Manitoba received a budgetary allocation of $72,437,000 in 2022 from the Federal Government towards clearing surgical backlogs.<br>**Reported Progress***<br>In January 2022, the Province reported a net increase of more than 9,000 procedures completed in 2021-22 including cataract, hernia, paediatric dental and spine surgeries.<br>The Diagnostic and Surgical Task Force Dashboard tracks<br>• wait times (median number of weeks)<br>• total number of patients who have been placed on a wait list, and<br>• completed cases as well as the progress made in addressing the pandemic backlog (where available).
**New Brunswick**<br>Stabilizing Health Care: An Urgent Call to Action (November 2021)	**Target**<br>• Eliminate long-waits (over a year) for hips and knees surgery, and reduce surgical wait times by 50 per cent by 2024<br>**Strategies**<br>• Implement surgical care pathways that reduce duration of hospital stay

	• Introduce electronic referral system that allows flexibility to choose next available specialist or wait for a specific surgeon<br>• Use e-consultation system to cut wait times and travel times for specialist care<br>**Funding**<br>New Brunswick received $41,238,000 from the federal budget for 2022 to clear surgical backlogs
**Newfoundland and Labrador**	Established Surgical Task Force in June 2022 to address surgical backlogs based on recommendations of the Newfoundland and Labrador Medical Association
**Nova Scotia**<br>○ Action for Health: A Strategic Plan 2022 – 2026<br>○ Surgical Access and Quality Improvement Strategy (May 2022)	**Goals**<br>• Support post pandemic recovery, stabilize surgical activity and volumes<br>• Achieve and sustain a reasonable surgical wait list<br>• Meet established benchmarks (surgeon defined priority and/or national benchmarks)<br>• Enhance patient-centered care<br>• Improve surgical outcomes<br>**Target (2022) -**<br>Reduce waitlist by completing 2,500 additional surgeries in the next 12 months.<br>**Strategies**<br>Multi-pronged approach focused on:<br>• Increasing capacity - increasing OR capacity, expand same-day surgery, ensure appropriate setting for procedures, have 'protected' surgical beds<br>• Increasing efficiency - single-entry referral model, centralized booking, more efficient OR utilization, reduce length of stay, OR allocation<br>• Managing demand - send demand to other sites with latent system capacity, wait list clean-up, appropriateness for surgery (shared decision-making), public-private partnerships<br>• Quality - Implementation of Society of Thoracic Surgery QI Registry, adherence to National Surgical Quality Improvement Program informed evidence-based toolkits, reduce variation in clinical standardization<br>**Funding**<br>• The Provincial Budget for 2021-22 allocated an increase of $1.9 million for orthopedic surgeries, to ensure more Nova Scotians can get hip and knee surgeries<br>• Budget 2022-2023 provided for $17.5 million to perform 2,500 more surgeries, expand operating room hours and add 28 beds and staffing at Dartmouth General Hospital to support more surgeries, $2.1 million to address surgery backlogs due to COVID-19 at IWK Health Centre and to fund more cataract surgeries and $597,000 to expand operating room capacity in Cape Breton<br>• Nova Scotia was awarded $51,800,000 in 2022 from the Federal Government towards clearing surgical backlogs.

	**Reported progress***<br>Metrics - % completion, other reporting metrics to be defined.<br>As of October 2022, the province had reached their pre-pandemic surgical volume (functioning at 101% of baseline capacity) b
**Ontario**<br>• Fall Preparedness Plan (July 2020)<br>• Surgical Recovery Plan (July 2021)<br>• Plan to stay open: health system stability and recovery (March 2022)	**Goal** – Increase surgical capacity by 110 to 115% to clear surgical backlogs<br>**Strategies**<br>• Provide funding for additional surgeries (including cancer, cardiac, cataract, and orthopaedic procedures) to take place during extended hours, and additional diagnostic imaging hours<br>• Address backlog through innovative channels such as the use of alternate health facilities that can deliver additional publicly funded surgical and diagnostic imaging services<br>• Maintain current operations for hospital capacity and add beds as needed to support increased surgical activity in hospitals<br>• Implement innovative solutions to address the surgical backlog: initiate a centralized waitlist management and a program to optimize the use of the operating rooms; improve the use of existing resources; increase the number of surgical procedures in hospitals by hundreds on average per year; and improve patient flow by leveraging available surgical capacity in each region<br>• Improve capacity for long-term and community care to ensure transition to more appropriate care options post-surgery, and allow for maximal use of surgical beds<br>**Funding**<br>• As part of the province's Fall Preparedness Plan (2020), the government invested $283.7 million to support additional priority surgeries and procedures<br>• In 2021, the provincial government invested $300 million to improve surgical and diagnostic capacity and cover up to 33,000 new cataract surgeries, 4,300 new orthopedic surgeries (e.g., hip replacement surgery) and 9,000 new pediatric surgeries. (Breakdown: $216 million for hospitals to extend operating room hours and perform up to 67,000 additional surgeries on top of the typical volume of 650,000; $35 million for additional CT and MRI operational hours; $18 million in centralized surgical waitlist management; $1 million for surgical smoothing coaching from an expert team of experienced surgeons and administrators; $30 million for the new Surgical Innovation Fund.)<br>• An additional funding of up to $24 million was provided to increase surgeries at existing and license new independent health facilities for services in 2021.<br>• By 2022, the government announced it was investing over $300 million in 2022–23 as part of the province's surgical recovery strategy, bringing the total investment to approximately $880 million over the last three fiscal years.

	• The province was allocated $775,500,000 in the 2022 Federal Budget to address surgical backlogs.<br>**Reported progress***<br>As of July 2021, Ontario Government reported that 76 per cent of patients who were waitlisted for required surgery between March 1, 2020, and March 1, 2021, have received the care they need. Throughout the pandemic, urgent surgical patients were prioritized and 99.3 per cent of the most urgent patient surgeries were completed.
**Quebec**<br>Continuation of the Strategy for the resumption of surgeries in Quebec (June 2021)	**Targets**<br>• Today: restore a 100% surgical activity rate (as of June 2021)<br>• October 2021: increase surgical activity rate to 115%<br>• March 2023: reduce the waiting list below the pre-pandemic level and significantly reduce the list of people who have been waiting for more than a year<br>**Strategies:**<br>• Review of waiting list<br>• Optimizing and increasing the use of operating rooms<br>• Addition of financial and material resources<br>• Close monitoring of the progress of surgeries and the reduction of waiting times<br>**Funding:**<br>• The province was allocated $405,006,000 in the 2022 Federal Budget to address surgical backlogs.
**Saskatchewan**<br>Target to Address Surgical Backlog and ICU Capacity (December 2021)<br>Ministry of Health Business Plan 2022-23	**Targets**<br>• Eliminate the COVID-related surgical backlog by 2025 and achieve three-month surgical wait time by 2030.<br>• Perform an additional 7,000 surgeries in 2022-23 over pre-pandemic levels. Volume targets will grow by an additional 6,000 in 2023-24 and 5,000 in 2024-25<br>• By March 31, 2023, 90% of surgical patients receive a first offer of surgery within 12 months and no patients are on the wait list longer than 24 months.<br>• By March 31, 2023, 90% of urgent cancer surgery patients receive a first offer of surgery within three weeks.<br>• By March 31, 2023, increase number of surgeries performed in Saskatchewan to 97,000.<br>**Strategies**<br>Incremental steps to achieve higher volumes of surgeries by implementing the following actions:<br>• Increase Saskatchewan Health Authority (SHA) capacity – expand and optimize operating room hours and making greater use of regional surgical sites; upskill and provide specialized

	training for registered nurses; prioritize surgical procedures with a higher number of long waiting patients.<br>• Increase private provider capacity – The ministry and SHA are working with our current third party contracted provider to increase the number and types of surgeries they perform and expand hours of operation.<br>• Request for Information – Issue a Request for Information for additional third-party surgical providers to achieve expanded day procedures, in-patient procedures, and additional capacity for post-operative care.<br>**Funding**<br>• Saskatchewan Government included a $21.6 million increase in Budget 2022 to address surgical wait lists<br>• The province received $61,759,000 from the Federal Budget to address surgical backlogs in 2022<br>**Reported Progress***<br>In June 2022, the Government of Saskatchewan reported that during the first five months of the year:<br>• The health system delivered 5,000 more surgeries than during the preceding five months in 2021 when services had been slowed down.<br>• There was a 2,500 (50%) increase in the number of surgeries performed on patients who had already waited over six months compared with the preceding five months in 2021.<br>• Saskatchewan hospitals had performed 240 more joint replacements during the first five months of 2022 compared with the same period in 2019, the last year without pandemic service disruptions.

*Progress as reported in provincial policy documents or official news updates
[a] Modelling estimates by the AHS were used to determine 2022/2023 targets
[b] Verbal report from provincial surgical leader
